# Supplementary material for: Intrinsic Myogenic Potential of Skeletal Muscle-Derived Pericytes from Patients with Myotonic Dystrophy Type 1
Source: Mol Ther Methods Clin Dev. 2019 Sep 12;15:120–32. doi: 10.1016/j.omtm.2019.09.002 (PMC6804802; doi:10.1016/j.omtm.2019.09.002)
Supplement: Document S1: Figures S1–S3 and Table S1 [file mmc1.pdf]

OMTM, Volume *15*

Supplemental Information

Intrinsic Myogenic Potential of Skeletal Muscle-Derived Pericytes from Patients with Myotonic Dystrophy Type 1

Cornelia Rosanne Maria Ausems, Renée Henrica Lamberta Raaijmakers, Walterus Johannes Antonius Adriana van den Broek, Marieke Willemse, Baziel Gerardus Maria van Engelen, Derick Gert Wansink, and Hans van Bokhoven

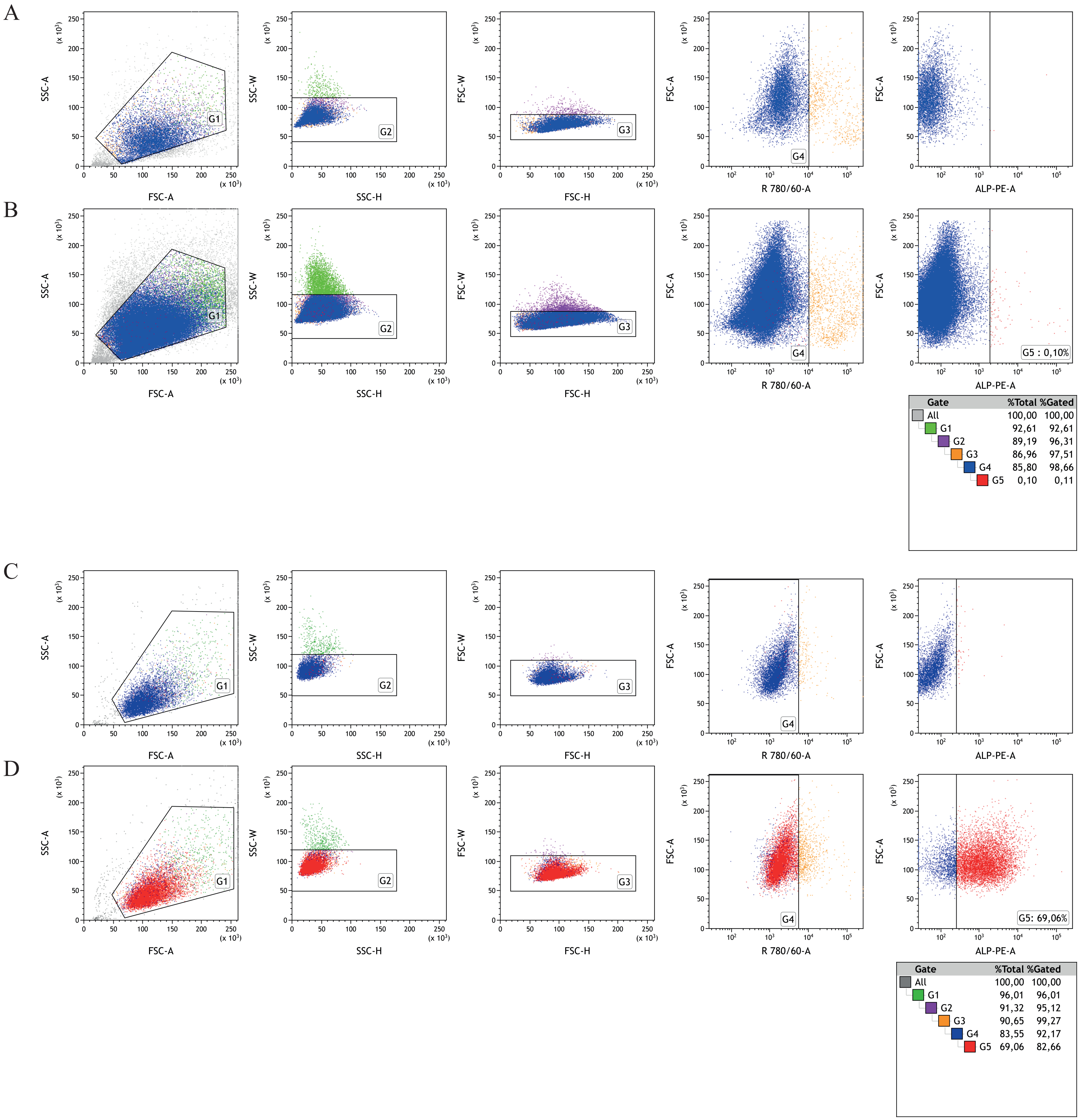

Figure S1. Gating boundaries and representative sorting of ALP$^+$ pericytes from mouse and human explant cultures. Harvested cells from the explant cultures were sorted on ALP$^+$. Cells in the fifth gate "G5" were considered ALP$^+$ pericytes. The population hierarchy is shown under the plots. (A) Gating for all mouse samples was optimized according to a negative control without ALP$^-$ phycoerythrin (PE). (B) Fluorescent plot of the sorting of mouse sample (M3), showing the distribution of the ALP-PE$^+$ pericytes. (C) Gating for all human samples was optimized according to a negative control. (D) Fluorescent plot of the sorting of ALP-PE$^+$ pericytes from the explant culture of P3. This supplemental figure relates to Figures 1 and 2.

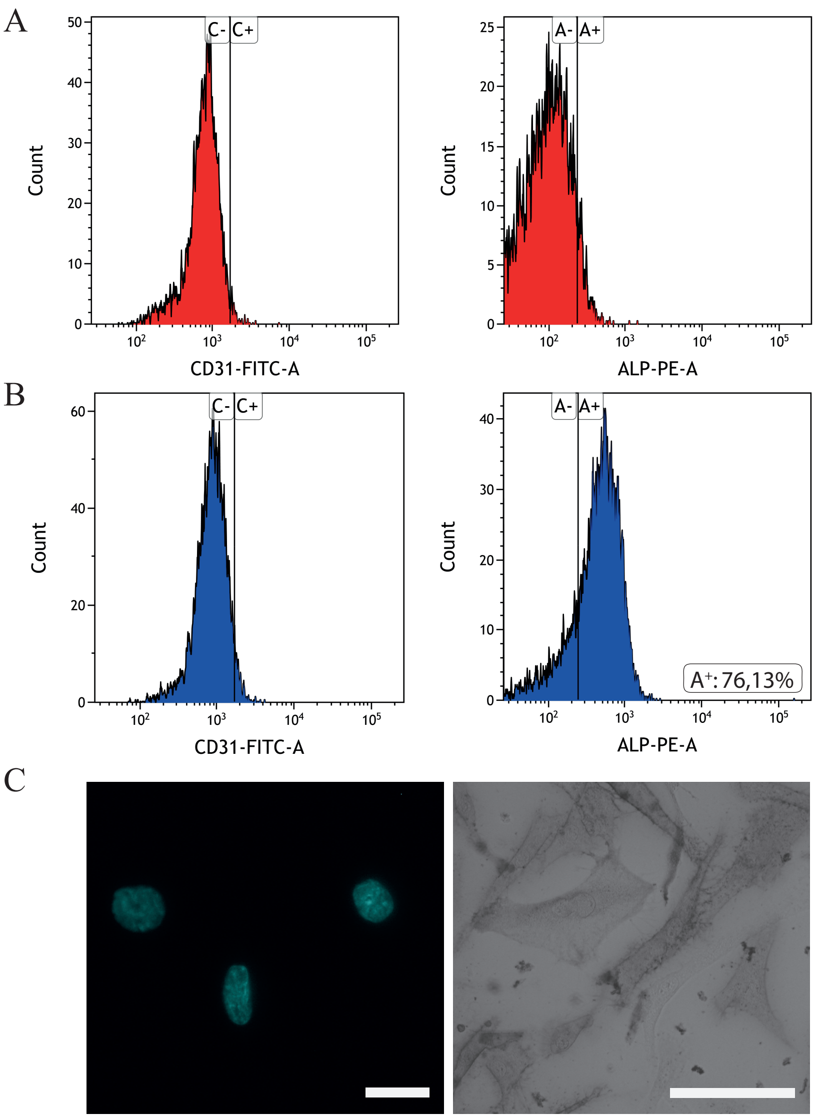

Figure S2. Determination of ALP expression in mouse and human pericytes after high cell passage number. Cell numbers of mouse pericytes plotted against $CD31^-$ fluorescein isothiocyanate (FITC) and ALP-PE staining. The input was a gated population without debris, cell doublets and dead cells (G4) (see Fig. S1B). A negative control without ALP-PE (A) was included to set the gate on background signal. The cell population remained ALP^+ after 19 passages (B). (C) Over time the culture of P6 showed stretched elongated cells. To verify whether at passage 20 the culture had started to differentiate, we stained for MHC expression (left) and ALP activity (right). All cells turned out to be MHC negative and ALP positive. Scale bars are 20 µm (left) and 100 µm (right). This supplemental figure relates to Figures 1 and 2.

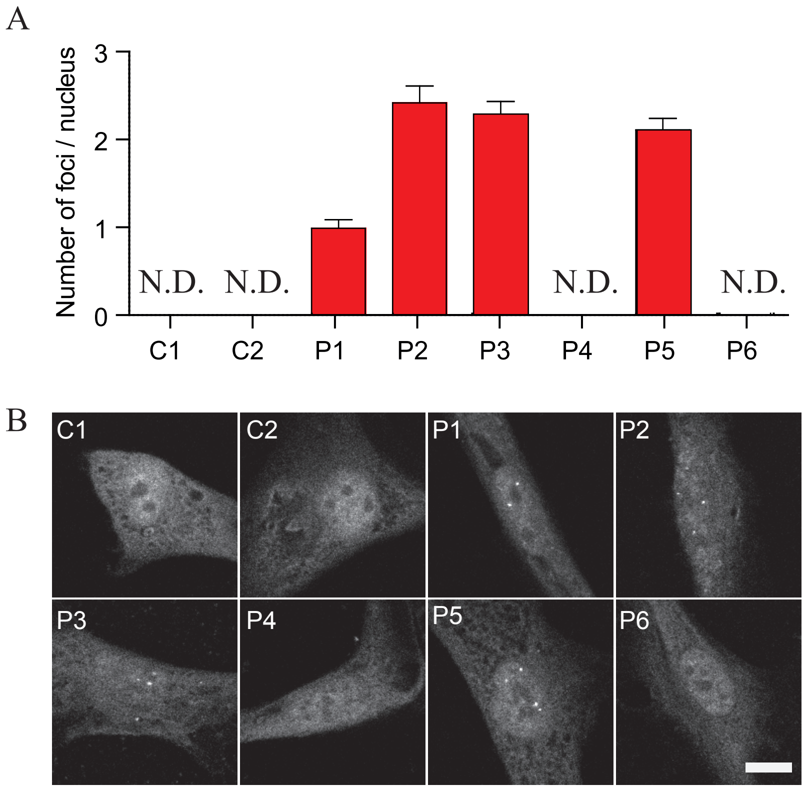

Figure S3. Representative RNA FISH images using a (CAG)7 oligonucleotide probe. Quantification of nuclear (CUG)n foci in pericytes isolated from two unaffected controls (C1, C2) and six DM1 patients (P1-P6). N.D. Not detectable. Scale bar is 10 µm. Error bars indicate SEM. This supplemental figure relates to Figure 6.

Table S1: Passage number and fusion index of pericyte cultures in myogenic differentiation experiments.

Participant number	Passage number experiment 1	Passage number experiment 2	MFI (%) experiment 1	MFI (%) experiment 2
C1	P04	P09	8.8	3.4
C2	P03	P05	2.8	10.5
P1	P01	P05	4.6	4.7
P2	P03	P04	1.9	1.7
P3	P02	P04	5.4	4.2
P4	P04	P04	1.6	1.6
P5	P04	P07	3.0	2.8
P6	P09	P12	14.5	9.6

MFI: myogenic fusion index
