# Supplementary material for: Intrinsic Myogenic Potential of Skeletal Muscle-Derived Pericytes from Patients with Myotonic Dystrophy Type 1
Source: Mol Ther Methods Clin Dev. 2019 Sep 12;15:120–32. doi: 10.1016/j.omtm.2019.09.002 (PMC6804802; doi:10.1016/j.omtm.2019.09.002)
Supplement: Document S2: Article plus Supplemental Information [file mmc2.pdf]

Molecular Therapy
Methods & Clinical Development
Original Article

Intrinsic Myogenic Potential of Skeletal Muscle-Derived Pericytes from Patients with Myotonic Dystrophy Type 1

Cornelia Rosanne Maria Ausems,[1,2,3] Renée Henrica Lamberta Raaijmakers,[1,2,3] Walterus Johannes Antonius Adriana van den Broek,[3] Marieke Willemse,[3] Baziel Gerardus Maria van Engelen,[2,4] Derick Gert Wansink,[3,4] and Hans van Bokhoven[1,4]

[1]Department of Human Genetics, Radboud University Medical Center, Donders Institute for Brain Cognition and Behavior, 6525 GA Nijmegen, the Netherlands; [2]Department of Neurology, Radboud University Medical Center, Donders Institute for Brain Cognition and Behavior, 6500 HB Nijmegen, the Netherlands; [3]Department of Cell Biology, Radboud University Medical Center, Radboud Institute for Molecular Life Sciences, 6525 GA Nijmegen, the Netherlands

Pericytes are multipotent, vessel-associated progenitors that exhibit high proliferative capacity, can cross the blood-muscle barrier, and have the ability to home to muscle tissue and contribute to myogenesis. Consequently, pericyte-based therapies hold great promise for muscular dystrophies. A complex multi-system disorder exhibiting muscular dystrophy for which pericytes might be a valuable cell source is myotonic dystrophy type 1 (DM1). DM1 is caused by an unstable (CTG)n repeat in the *DMPK* gene and characterized by skeletal muscle weakness, muscle wasting, and myotonia. We have successfully isolated alkaline phosphatase-positive pericytes from skeletal muscle of DM1 patients and a transgenic mouse model. Intranuclear (CUG)n RNA foci, a pathogenic DM1 hallmark, were identified in human and mouse pericytes. Notably, pericytes from DM1 patients maintained similar growth parameters and innate myogenic characteristics *in vitro* compared to cells from unaffected controls. Our *in vitro* results thus demonstrate the potential of pericytes to ameliorate muscle features in DM1 in a therapeutic setting.

INTRODUCTION

In a search for myogenic cells from non-somite origin, De Angelis and colleagues were the first to show that vessel-associated cells isolated from the wall of the embryonic dorsal aorta, generally called mesoangioblasts, hold myogenic potential.[1,2] Follow-up research revealed that their postnatal analogs surrounding vessels in postnatal tissues, pericytes, also have the capacity to contribute to myogenesis.[3] The close association of pericytes with the vasculature positions the cells at the forefront of endogenous tissue repair responses. Consequently, pericytes seem to be indispensable for growth and regeneration of muscle fibers during postnatal life.[4–6]

Isolation of pericytes can provide researchers with a promising pool of undifferentiated cells capable of differentiating into other tissue types when experimentally relocated.[7] Encouraging results in the field of Duchenne muscular dystrophy (DMD),[8] limb-girdle muscular dystrophy (LGMD),[9] and facioscapulohumeral muscular dystrophy (FSHD)[10] made us wonder whether functional pericytes are also present in postnatal tissues of patients with the variable and most prevalent neuromuscular disease, myotonic dystrophy type 1 (DM1). If so, would these pericytes present DM1 hallmarks and still hold myogenic potential? In fact, it has been reported that pericyte abundance is negatively correlated with increasing age due to an age-related decrease in the microvasculature system of the skeletal muscle.[11] Because DM1 is considered to be a progeroid syndrome with progressive age-dependent dysfunction of many organ systems,[12,13] an increased demand of progenitor cells might exhaust the pericyte cell pool. Additionally, pericytes isolated from elderly subjects, and consequently DM1 patients, could be impaired in proliferation and myogenic differentiation ability.[14]

DM1 is a multisystemic repeat expansion disorder with pathogenic effects brought about by an expanded (CTG•CAG)n repeat in the *DMPK/DM1-AS* gene pair.[15–17] Because this repeat tends to show somatic and intergenerational instability, DM1 is one of the most variable genetic diseases.[18,19] An increase in repeat length, from 50 up to a few thousand triplets, correlates with more severe symptoms and an earlier age of onset. Expression of expanded *DMPK* RNA causes sequestration of RNA binding proteins (RBPs), such as members of the muscle blind-like family (MBNL) of proteins. Formation of these ribonuclear complexes, visualized as so-called foci in microscopy, is thought to initiate a cascade of downstream effects resulting

Received 6 June 2019; accepted 4 September 2019;
https://doi.org/10.1016/j.omtm.2019.09.002.

[4]These authors contributed equally to this work.

Correspondence: Hans van Bokhoven, Department of Human Genetics, Radboud University Medical Center, Donders Institute for Brain Cognition and Behavior, Geert Grooteplein 10, 6525 GA Nijmegen, the Netherlands.
E-mail: hans.vanbokhoven@radboudumc.nl
Correspondence: Derick Gert Wansink, Department of Cell Biology, Radboud University Medical Center, Radboud Institute for Molecular Life Sciences, Geert Grooteplein 26, 6525 GA Nijmegen, the Netherlands.
E-mail: rick.wansink@radboudumc.nl

 © 2019 The Author(s).
This is an open access article under the CC BY license (http://creativecommons.org/licenses/by/4.0/).

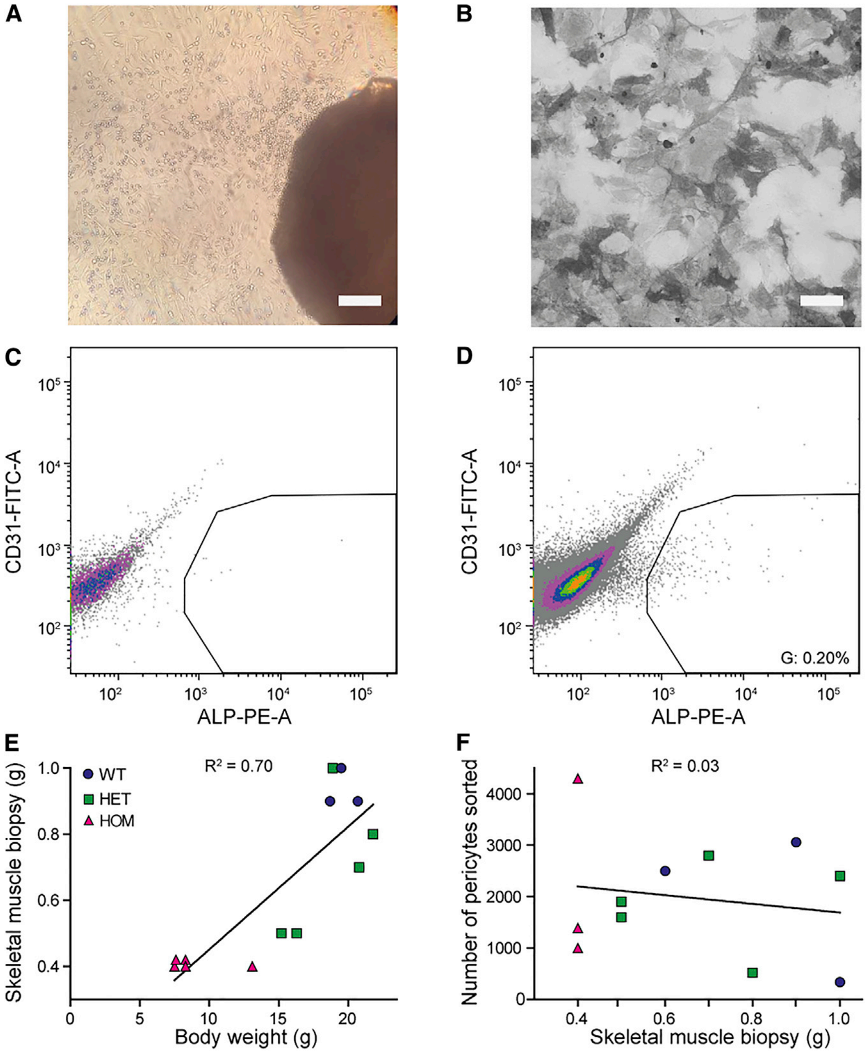

Figure 1. Isolation of Pericytes from DMSXL Mice and WT Controls

(A) Outgrowth of cells (left top corner) from a skeletal muscle explant (right bottom corner) of a DMSXL mouse, 7 days after muscle isolation. Scale bar, 300 μm. (B) ALP-activity staining on sorted pericytes. Scale bar, 100 μm. (C) Representative flow cytometry analysis of a negative control for surface expression of ALP. Colors represent a density plot. Gates were set according to the negative control. (D) Representative flow cytometry analysis of sample. The ALP^+ results are between 0.1% and 0.4% of the total unsorted population. Input is live cells without debris or doublets (Figure S1B). (E) Relationship between the weight of muscle dissected from the hind limbs and whole-mouse body weight. Each data point represents data from one mouse (n = 3–5 per genotype). (F) The number of ALP^+ pericytes sorted from skeletal muscle biopsies after explant cultures. Each data point represents data from one mouse (n = 3–5 per genotype).

in widespread dysregulated RNA processing, including alternative splicing and polyadenylation.[15] Additionally, repeat-associated non-AUG (RAN) translation of repeat transcripts may contribute to disease via the production of toxic homopolymeric proteins.[20,21] Taken together, the expanded repeat results in a complex set of features in patients. For skeletal muscle this relates to progressive muscle weakness, muscle wasting, and myotonia. Currently, clinical management of DM1 patients is limited to symptomatic treatment.[22]

The myogenic cell type that is first harmed in DM1 by repeat-expanded RNA during development, and therefore must be repaired in cell-based therapeutic strategies, has not been identified. The onset of *DMPK* expression is already seen in somites in developing embryos, even before commitment to specific muscle cell fate and onset of myogenesis.[23,24] To investigate the potential of pericytes for therapeutic purposes, we attempted to isolate pericytes from patients with variable repeat lengths and DM1 mice. These pericytes were cultured *in vitro* and used for characterization of gene expression, cell growth, and myogenic fusion capacity. Spontaneous differentiation of human pericytes, triggered by serum reduction, indeed resulted in fused and elongated myosin heavy chain (MHC) positive multi-nucleated myotubes, without obvious differences between cells from patients and unaffected controls. Our results indicate that pericytes from skeletal muscle of DM1 patients and DMSXL mice may pave the road for cell therapy approaches.

RESULTS

Explant Cultures of Skeletal Muscle from DMSXL Mice and DM1 Patients

Culture of tissue fragments *in vitro*, hereafter called explants, can give rise to the outgrowth of a mixed population of cells. Due to the inability of other cell types to rapidly proliferate under the specific conditions used here, we expected a progressive increase in the proportion of pericytes. The isolation and characterization of mouse pericytes is depicted in Figure 1, while Figure 2 provides the results of the human muscle biopsies. Skeletal muscle was chosen as the source for our explant cultures since muscle-derived pericytes show a preference for differentiation into the myogenic lineage.[25–27] For the isolation of mouse pericytes, we collected skeletal muscle tissue of the hind limbs of DMSXL mice. These mice express a human DM1 allele including a CTG1300 repeat.[28,29] Regulatory sequences from the human locus drive a near-to-natural expression of these transgenes in all DM1-related tissues (skeletal muscle, heart, CNS), making it a valuable and useful model to study DM1 pathomechanisms.[30] Within a few days, a mixed population of cells grew out from dissected small mouse muscle tissue fragments (Figure 1A).

For the isolation of human pericytes, we included six DM1 patients and two unaffected individuals as controls. The participants showed

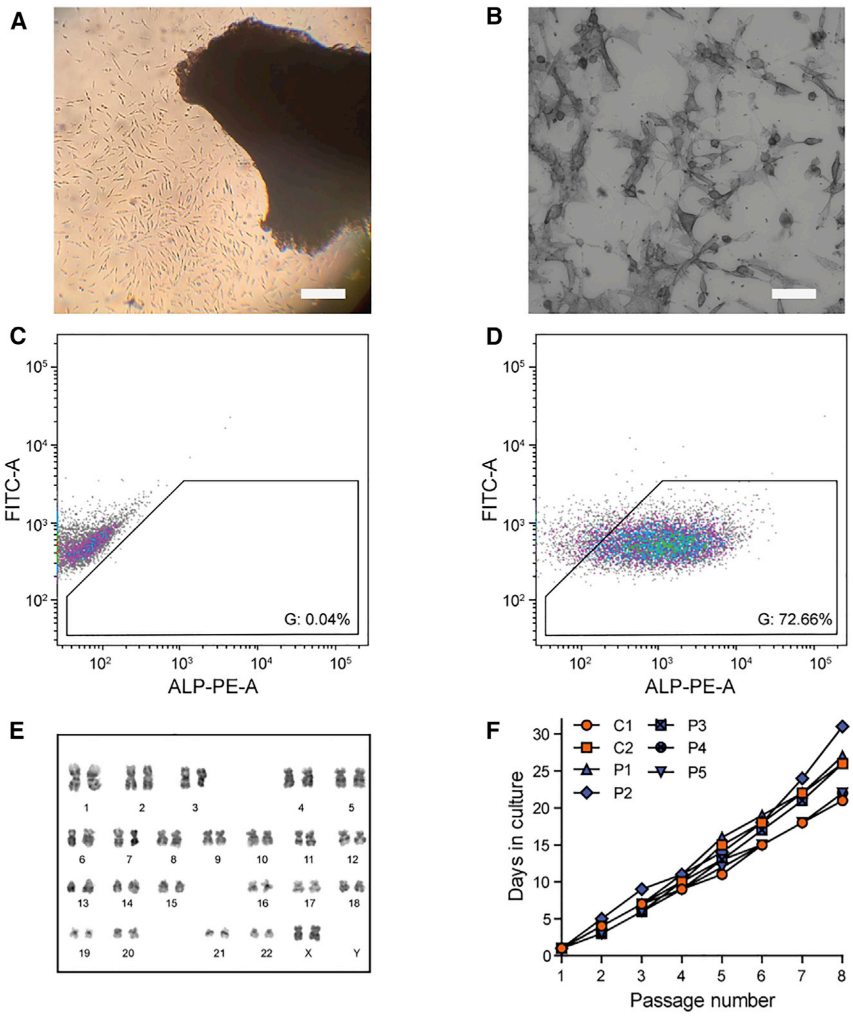

Figure 2. Isolation of Pericytes from DM1 Patients and Unaffected Controls

(A) Outgrowth of cells from a quadriceps muscle explant. Scale bar, 300 μm. (B) ALP-activity staining on sorted pericytes. Scale bar, 100 μm (C) Representative flow cytometry analysis of negative control. Colors represent a density plot. Gates are set according to the negative control. (D) Representative flow cytometry analysis of sample. Sorting results are high due to the continued selection of pericytes via replating of cells. Input is live cells without debris or doublets (Figure S1D). (E) Karyogram of pericytes from P6 after nine passages, showing a euploid number of chromosomes. (F) Growth curves of human pericyte cultures, starting from the first passage of purified pericytes. Cells were split 1:4 at each passage.

a broad diversity in age and sex (Table 1). The DM1 patients had variable (CTG)n repeat lengths and scores on the Muscular Impairment Rating Scale (MIRS), a simple and reliable five-point rating scale for muscular impairment in DM1.[31] Muscle biopsies from the quadriceps were cultured following the same approach as for mouse tissue. Initially, the human explant cultures showed increased presence of red blood cells. Repeated removal of these small nonadherent cells via selected washing and replating of the explants was necessary to promote efficient outgrowth of adherent cells (Figure 2A).

Purification and Validation of Mouse and Human DM1 Pericytes

Pericytes can be identified by the presence of alkaline phosphatase (ALP).[3,6,32] In contrast to other pericyte markers such as neuron-glial antigen 2 (*NG2*) and platelet-derived growth factor receptor beta (*PDGFRβ*), *ALP* is not expressed in skeletal muscle fibers, nor in other myogenic progenitors, but it is restricted to the microvasculature of striated muscle in postnatal life[6] and is therefore an appropriate selection marker. Expression of this phosphatase by pericytes enabled us to distinguish them from PAX7$^+$ or MYOD$^+$ satellite cells, which might also be present in the explant cultures. Moreover, pericytes lack endothelial markers such as CD31.[3]

To obtain ALP$^+$ cells from the mixed population of outgrown mouse cells, we sorted these cells via fluorescent-activated cell sorting (FACS) on the presence of ALP and absence of CD31 on day 7 (Figures 1C and 1D; Figures S1A and S1B). Enzymatic ALP staining in all cells after sorting confirmed our selection protocol (Figure 1B). Due to the presence of blood cells in the human cultures, it took 7 days longer for an outgrowth ring of cells to appear. Cell sorting of five cell lines (control-derived lines C1 and C2, and patient-derived lines P1, P3, P6) showed that via replating under pericyte-favorable conditions, we had already established a selection for ALP$^+$ and CD31$^-$ cells during cell culture (Figures 2C and 2D; Figures S1C and S1D). Consequently, the last three patient-derived lines (P2, P4, P5) were not sorted but were validated via enzymatic ALP stain (Figure 2B). We thus were able to collect pure ALP$^+$ cultures from all participants (Table 1).

After sorting of mouse and human ALP$^+$ cells, we further confirmed the cell type via immunocytochemistry. A combination of pericyte markers alpha smooth muscle actin (α-SMA), NG2, and PDGFRβ, combined with absence of MHC, clearly demonstrated both identity and purity of our pericyte isolates from both mouse and human muscle (Figure 3).

Homozygous (HOM) DMSXL mice show high neonatal mortality, muscle defects, and growth retardation, whereas heterozygous (HET) mice show milder muscle symptoms and no increase in neonatal death.[29] As expected from these phenotypes, we found a strong positive correlation (R^2 = 0.70) between body weight of the mice and the amount of skeletal muscle isolated from the hind limbs (Figure 1E). Unexpectedly, however, no correlation existed between

Table 1. Clinical and Genetic Data of Included Participants

Participant No.	Sex (M/F)	Disease (CONT/ DM1)	MIRS	Repeat Length at Diagnosis (No. of Triplets)	Age at Biopsy (years)
C1	M	CONT	NA	NA	45
C2	F	CONT	NA	NA	22
P1	M	DM1	5	120 < N < 1000	46
P2	F	DM1	4	>200	50
P3	F	DM1	4	>200	39
P4	M	DM1	3	50–150	71
P5	F	DM1	3	>150	51
P6	F	DM1	2	52	29

CONT, unaffected control; DM1, DM1 patient; M, male; F, female; NA, not applicable.

the total weight of the muscle biopsy used for the explant cultures and the number of pericytes obtained after sorting ($R^2 = 0.03$; Figure 1F), and comparable numbers of pericytes could be isolated from HOM, HET, and wild-type (WT) mice. Importantly, we cannot exclude that the abundance of pericytes in skeletal muscle tissue *in vivo* varies between the genotypes.

Human pericytes maintained their diploid karyotype after at least nine passages (Figure 2E) and showed a typical triangular refractile morphology for at least 20 population doublings, with a doubling time of approximately 40 h (Figure 2F). Notably, the proliferation rate of all pericyte cultures was comparable and independent of donor, MIRS, sex, and participants' age (note that no growth curve was measured for P6, as this biopsy was used to optimize the isolation protocol).

To examine the progenitor cell state at higher passages, we re-sorted mouse pericyte cultures at passage 19. The entire population of cells still expressed *Alp* (Figures S2A and S2B), so no differentiation toward myoblasts or contamination of additional cell types was encountered. For human pericytes, we performed MHC staining at passage 20 to verify that they did not differentiate toward MHC^+ myotubes. MHC staining was negative and all cells still expressed *ALP* (Figure S2C).

Characterization of Disease Miomarkers in DM1 Pericytes

The DM1 mutation is an autosomal-dominant, unstable (CTG)n repeat in the 3′ untranslated region of *DMPK*. The repeat mutation ends up as a long (CUG)n stretch in pathogenic, expanded transcripts, which initiate a broad range of detrimental downstream effects.[33] We investigated the expression of endogenous *Dmpk* and transgenic *DMPK* transcripts in pericytes from the DMSXL line. *Dmpk* expression in proliferating pericytes was similar between cells isolated from WT, HET, and HOM mice, as well as DM500 myoblasts (Figure 4A). DM500 myoblasts are an immortalized cell lineage that was isolated earlier from the same transgenic mouse line, when the (CTG)n repeat still contained only ~500 triplets.[17,28] No significant differences were found in *DMPK* (CTG)1300 transcript levels between HET and HOM pericytes, carrying one and two transgene copies, respectively. As expected, no *DMPK* expression was detected in WT pericytes (Figure 4B). *DMPK* expression in human pericytes was remarkably similar between patients and controls, and also comparable to that in human myoblasts (Figure 4C).

According to the RNA dominance theory, expression of *DMPK* RNA with abnormal repeat length may affect proper muscle development and have consequences for regenerative muscle capacity in adulthood.[34] Both the timing and the level of expression of pathogenic repeats will influence the type and the extent of down-stream toxicity caused by for example RNP binding or RAN translation products.[17,35] Pericytes isolated from HET and HOM DMSXL mice displayed expanded *DMPK* transcripts in the form of fluorescent *in situ* hybridization (FISH)-detectable RNP complexes in the nucleus, generally termed foci, after hybridization with a CAG repeat probe. The number of foci varied strongly between cells, but, on average, 1–2 foci per nucleus were present in pericytes from HET mice versus 5–10 foci in cells from HOM mice (Figure 5). Few foci were detected in some WT mouse cells, which has been reported before for myoblasts and may represent background staining recorded by the automated image analysis.[17] Especially noticeable was the high variation in foci count in pericytes from HOM animals, ranging between 0 and >25 per nucleus.

We also examined the occurrence of FISH foci in human pericytes. Pericytes from P1, P2, P3, and P5 showed an average of 1–2 foci per nucleus. No foci could be detected in pericytes from P4 and P6, presumably due to the smaller repeat lengths, which reduces the number of fluorescent probes that can bind to one transcript. As expected, no foci were detected in control pericytes (Figure 6; Figure S3). To verify MBNL1 protein accumulation in the (CUG)n RNA foci, we combined FISH with immunofluorescent detection of MBNL1. Nuclei in DM1 patient pericytes, but not controls, consistently showed focal accumulation of MBNL1 protein, which colocalized with (CUG)n RNA FISH foci (Figure 6). The number of MBNL foci counted per nucleus was slightly lower than the number of FISH foci, which is probably due to automated image analysis and thresholding.

DM1 Patient Pericytes Maintain Intrinsic Myogenic Potential

To examine whether DM1 pericytes still contained intrinsic myogenic potential, we focused on the purified human cells, since isolated mouse pericytes need co-culture with a myogenic inducer cell line for myotube formation *in vitro*.[11,36] Human pericytes were grown to confluency and then shifted to low-serum conditions to initiate myogenic commitment and fusion to myotubes (Table S1). *MHC* expression was used as a differentiation marker.[3] Notably, we were able to obtain MHC^+ fused and elongated myotubes from all control and patient pericyte populations. Up to 1.5-mm-long myotubes containing multiple nuclei were seen after keeping pericytes for 8 days under differentiation conditions (Figures 7A and 7B). Although strong variations between different isolates were observed, the differences in myogenic fusion efficiency could not be linked to disease state, sex, age of participant, or passage number of pericytes. Besides variations in fusion potential between individual cell cultures and

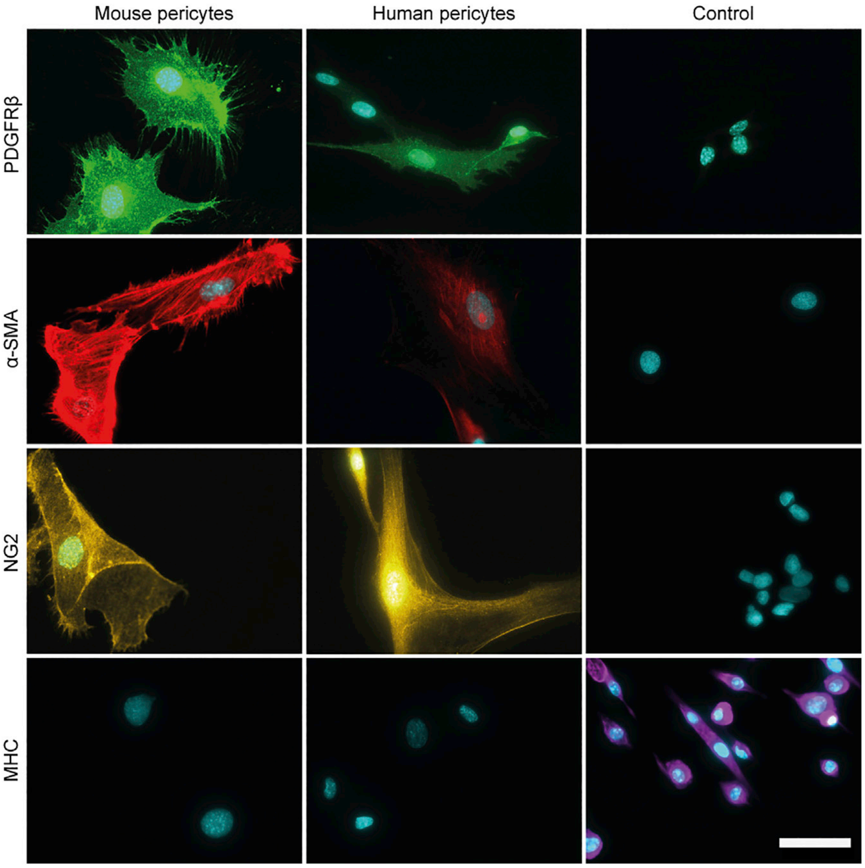

Figure 3. Validation of Purified Pericytes by a Combinatorial Staining Approach

Immunofluorescent staining of mouse and human pericytes for *Pdgfrβ/PDGFRβ*, *α-Sma/α-SMA*, and *Ng2/NG2* expression. *Mhc/MHC*, a marker for myogenic differentiation, was not expressed. Control cell lines: DMSXL myoblasts (*Pdgfrβ*), C25 human control myoblast (*α-SMA*), SH-SY5Y human cell line (*NG2*), and DMSXL myotubes (*Mhc*). Scale bar, 50 μm.

separate experiments, diverse myotube morphologies were generally observed in all cultures. Roundish or elongated MHC$^+$ mononuclear cells were visible (Figures 7C and 7D), and occasionally even mononucleated MHC$^+$ cells with sarcomeric striations were seen (Figure 7E).

To quantify myogenic differentiation, we defined myotubes as MHC$^+$ cells containing at least two nuclei (Figures 7F and 7G), and we determined the myogenic fusion index (MFI; 100 × the ratio of the number of nuclei inside MHC$^+$ myotubes to the number of total nuclei), the number of nuclei per myotube, and the myotube width and length (Figures 7H–7K). The MFI was variable between cultures (average, 5.3% ± 4.4%), and also the number of nuclei per tube (3.6 ± 2.6) differed between donors. The myotube width and length were similar for all cultures (22.6 ± 8.5 μm and 422 ± 179 μm, respectively). However, under the conditions tested in this study, none of the four parameters showed a significant difference between control and patient pericyte populations, and therefore we conclude that DM1 pericytes still maintain normal, intrinsic myogenic capacity, independent of the disease state.

DISCUSSION

In this study, we generated explant cultures of skeletal muscle from DMSXL mice, DM1 patients, and unaffected controls. From these cultures, through a combination of FACS and immunocytochemistry, we purified and identified vessel-associated pericytes, a myogenic progenitor cell type that holds promise for cell-therapeutic purposes in muscular disorders. Mouse model and patient pericytes displayed typical DM1 disease characteristics, such as *DMPK* expression and nuclear repeat RNA foci, but still demonstrated great proliferative ability. We were able to show that, despite the presence of these disease biomarkers, human DM1 pericytes displayed normal myogenic capacity *in vitro*, which facilitates their therapeutic application.

In adult skeletal muscle, only vessels contain ALP$^+$ cells.[3,37] Since pericytes are ALP$^+$ and endothelial cells are ALP$^-$, we could isolate ALP$^+$ cells with myogenic potential, while excluding myoblasts and satellite cells from our cultures.[3,6] FACS sorting was validated via immunocytochemistry, by which we found around 1% ALP$^+$ mouse cells in our primary cell isolates. Because we followed a strict sorting protocol, the actual percentage of sorted mouse ALP$^+$ cells was lower. Similar numbers of ALP$^+$ cells were isolated out of different amounts of skeletal muscle from WT, HET, and HOM DMSXL mice. Presumably, since pericytes are wrapped around blood vessels, the amount of skeletal muscle has no effect on the number of pericytes present in the tissue, but the number of blood vessels does. Because it has been reported that the vasculature between slow and fast fiber types differs in rats, with slow oxidative muscle possessing a more enriched capillary structure than the fast glycolytic muscle[38], it would be interesting to investigate pericyte abundance within these tissues. We expect that our cell populations constitute a mix of pericytes originating from fast and slow muscle tissue in the gastrocnemius-plantaris-soleus biopsy. When translating to humans, the observation that comparable numbers of pericytes could be sorted from HOM mice and their WT littermates is promising for an autologous therapy for DM1 patients.

Six DM1 patients and two unaffected controls were included for isolation of human pericytes. No differences in the number of pericytes or in the proliferation capacity between sorted cells from DM1 patients and controls were found. The human pericytes were isolated from a

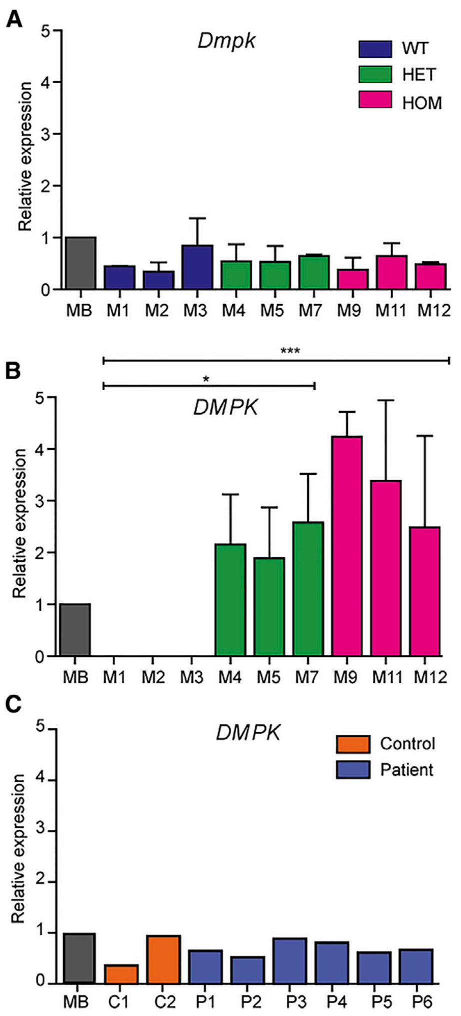

Figure 4. *Dmpk/DMPK* Expression in Mouse and Human Pericytes

qRT-PCR quantification of Dmpk and DMPK expression. Endogenous Dmpk (A) and transgenic DMPK (B) expression in pericytes from WT, HET, and HOM DMSXL mice. DMPK expression was significantly higher in HOM and HET pericytes compared to WT pericytes. Immortalized DM500 mouse myoblasts (MB) were included as reference (n = 2).[17] *p < 0.05, ***p < 0.0001. (C) DMPK expression in pericytes from unaffected controls and DM1 patients. Immortalized human DM1 myoblasts (MB) were included as reference (n = 1).[50] Error bars indicate SEM.

needle biopsy from the quadriceps muscle, a relatively spared proximal muscle in DM1 that usually is involved only in later, more severe stages of the disease.[34] We are unaware whether distal muscles marked by typical DM1-associated dystrophy show pericyte-related differences in abundance or proliferation capacity. Previous research showed a significant, 2-fold increase in the number of satellite cells in the distal *tibialis anticus* muscle compared to the proximal *vastus lateralis* muscle of the same DM1 patients.[39] The satellite cells isolated from the distal muscles, however, had a reduced proliferative capacity due to the abnormal activation of the p16 stress pathway, related to premature senescence.

A comprehensive verification of cell identity, based on a combination of phenotypic markers, validated that we isolated and expanded authentic pericytes from both mouse and human muscle. Pericyte markers may be dynamic in their expression and can be upregulated or downregulated in conjunction with developmental states, pericyte maturity,[40–42] pathological reactions, and *in vitro* culturing.[43,44] We found *α-Sma/α-SMA*, *Ng2/NG2*, and *Pdgfrβ/PDGFRβ* to be consistently expressed, while the myogenic marker *Mhc/MHC* was absent.

The prenatal onset of congenital DM1 implies a pathogenic effect of expanded *DMPK* alleles early during embryonic development.[35,45] Jansen et al.[23] noticed that *Dmpk* expression could be detected as early as embryonic day 10.5, before commitment to specific muscle cell fate and onset of myogenesis. Expression of normal and expanded *Dmpk/DMPK* transcripts is generally low and occurs at an absolute number of only a few to a few dozen transcripts per cell.[17] We found no obvious differences in expression of *Dmpk/DMPK* between pericytes from DMSXL genotypes nor in *DMPK* expression between pericytes from different patients with DM1 or unaffected controls. We did not separately quantify expanded *DMPK* transcripts from normal transcripts, but previous research has shown that both types of alleles contribute generally equally to *DMPK* RNA levels in human myoblast cultures and skeletal muscle from DM1 patients. Moreover, no evidence for a marked effect of repeat length on *DMPK* RNA expression level has been reported.[17]

It is not surprising that pericytes isolated from both HET and HOM DMSXL mice showed pathogenic transcripts as FISH-detectable foci in the nucleus. Similarly, pericytes from DM1 patients with a repeat above 200 triplets showed on average one to two foci per nucleus. Noticeable is the high variation in foci count in pericytes from HOM animals, ranging from 0 to up to >25 per nucleus, but these numbers match our observations regarding *DMPK* expression and foci number in myoblasts from this transgenic mouse line.[17] We have provided evidence that every RNP complex that forms a FISH-visible aggregate is nucleated by one or only few expanded (CUG)n transcripts.[17] Due to inaccessibility of the transcripts to the probe or loss of RNA molecules from the fixed cells during washing, the actual number of RNP complexes in the nucleus may even be a bit higher.[17,46] Due to technical differences between (CUG)n RNA FISH and immunofluorescence detection of MBNL1 protein, the quantification of MBNL1 foci resulted in lower foci number per nucleus. In all cases, however, (CUG)n RNA FISH foci colocalized with high concentrations of MBNL1 protein.

No major variations in myogenic capacity between pericytes from DM1 patients and unaffected controls were found. We were able to generate multinucleated MHC$^+$ myotubes from all human pericyte populations. Sometimes cultures showed mononuclear MHC$^+$ cells and myosacs, while others showed mainly textbook multinucleated and striated myotubes. We attribute these differences between cultures and experiments to subtle, not well-understood differences in culture conditions at the start of myogenic differentiation. For the quantification of MFI and myotube length and width, the presence

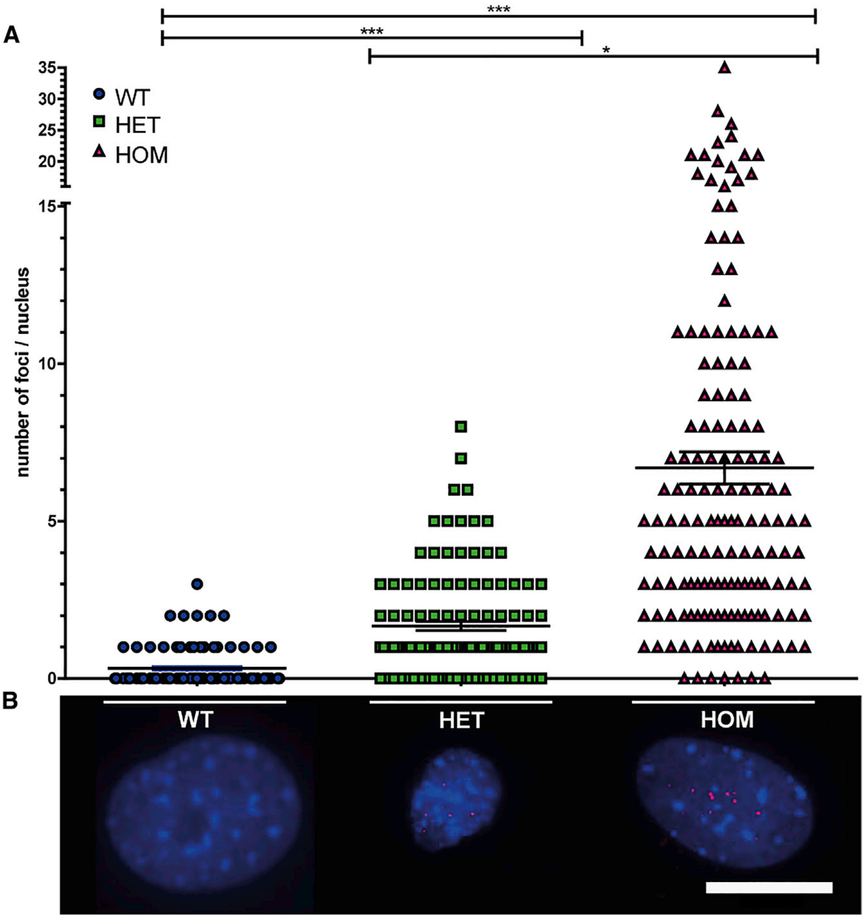

Figure 5. DM1 Foci in Transgenic Mouse Pericytes

DM1 foci were visualized by RNA *in situ* hybridization using a fluorescent (CAG)6 probe in mouse pericytes. (A) Quantification of the number of foci per nucleus for pericytes from WT, HET, and HOM mice. At least three mice per genotype were included, and at least 20 nuclei per mouse were analyzed with a minimum of 125 nuclei per genotype. Statistical analysis was performed between genotypes. There are significantly more foci present in pericytes derived from HET and HOM mice compared to pericytes from WT mice and significantly more foci are visualized in the HOM-derived pericytes compared to pericytes from HET mice. Data points represent the foci number per nucleus; the mean ± SEM is plotted in the graph. $^{*}p < 0.05$, $^{***}p < 0.0001$. (B) Representative RNA FISH images. Scale bar, 20 μm.

of mononucleated MHC+ cells and myosacs could have a great influence on the outcome. For the length and width quantifications, all mononuclear cells were excluded. Thin MHC+ mononucleated cells, which may be regarded as incompletely differentiated and immature muscle fiber growth,[34,47] affected MFI values.

Onset of disease and myopathy in DM1 are highly diverse, but clearly positively correlated with repeat length. We therefore expected the myogenic potential to decrease with an increased number of CTG triplets in *DMPK*. In fact, pericytes isolated from FSHD patients showed normal morphology and proliferation, but impaired differentiation correlated with overall disease severity.[48,49] Even though many variables differ between individuals in our diverse cohort, DM1 disease status, age, and sex did not influence isolation, proliferation, or differentiation abilities of pericytes. The observed variation in MFI might be partly explained by the genetic background of the individuals, but it is also regularly observed in the formation of myotubes from myoblasts.[50]

To obtain an even higher, and possibly more continuous, differentiation efficiency, pericytes can be exposed to a "myogenic environment," e.g., co-culture with murine myoblasts or direct reprogramming by overexpression of selected transcription factors such as MYOD. With MYOD induction, cells are pushed toward myogenic lineage and therefore exhibit an increased myogenic ability. However, this behavior does not necessarily correspond to normal fate during unperturbed development and regeneration.[51,52] Results from the present study show the innate myogenic ability of primary pericytes from DM1 patients.

The promise of pericytes for cellular therapy in muscular dystrophies is mainly due to the widespread distribution of these cells through the capillary network, a distinct advantage over previous cell-based approaches.[53] For example, pericytes that express a missing protein (dystrophin in DMD) or lack the RNA gain-of-function mutation (repeat expansion in DM1) may be systemically administered.[8,54,55] Autologous pericytes can infiltrate multiple target tissues from the circulation in response to cytokines released by dystrophic muscle. In the dystrophic target tissue, the healthy pericytes can replenish the progenitor pool and from then on efficiently differentiate toward the myogenic lineage and contribute to muscle regeneration by formation of healthy postmitotic myofibers. We were able to culture pericytes for up to 20 passages. Using *in vitro* expansion, we will be able to obtain 8.8×10^{16} cells from approximately 8×10^4 culture-initiating cells, a promising number for future therapeutic applications when compared on a per kilogram comparison with previously used animal models.[8,54]

So far, research on the presence and potential of pericytes in DM1 was missing. Several successful attempts to treat other muscular dystrophies with vessel-associated pericytes in mice models have been performed.[9,54] A translational study in a dog model of DMD myopathy showed "a remarkable clinical amelioration and preservation of active motility."[8] The first exploratory clinical trial for DMD investigated the safety of intra-arterial transplantation of HLA-matched donor

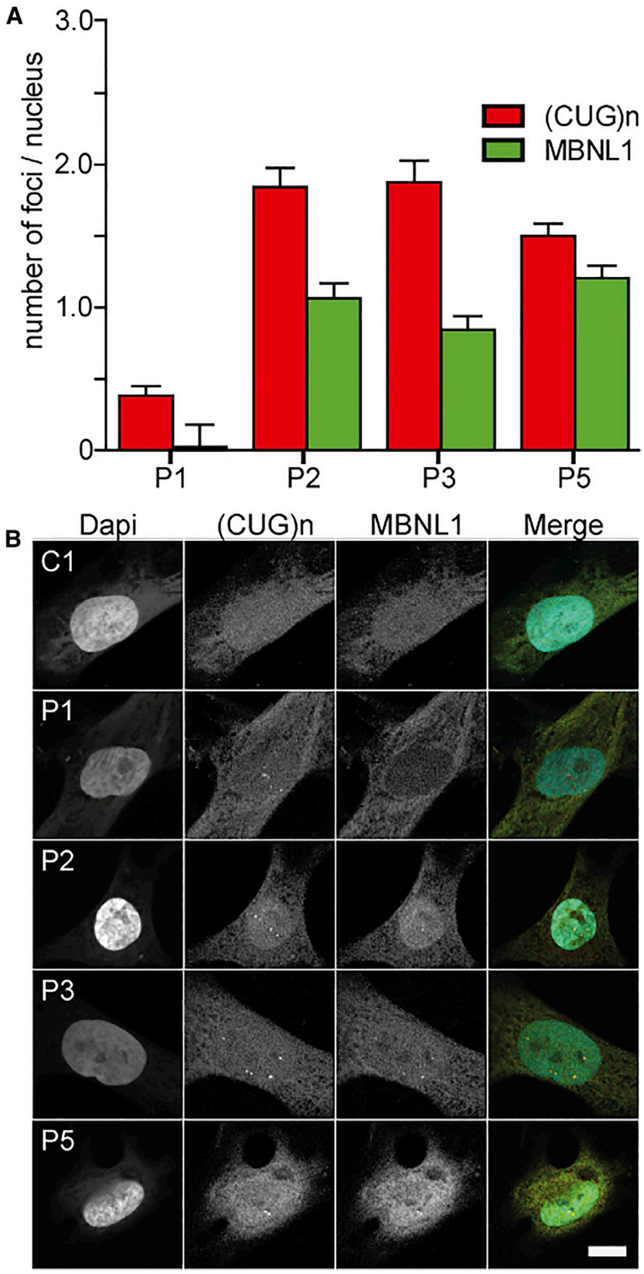

Figure 6. DM1 Foci in DM1 Patient Pericytes

(A) Automated quantification of nuclear (CUG)n and MBNL1 foci for P1, P2, P3, and P5. At least 60 nuclei per pericyte population were analyzed. (B) Simultaneous detection of DM1 foci by RNA *in situ* hybridization using a fluorescent (CAG)6 probe and immunofluorescence using a MBNL1 antibody in human pericytes. Error bars indicate SEM. Scale bar, 10 μm.

pericytes.[55] Based on the results presented herein, we will now work toward autologous and possibly genetically edited[50] pericytes that can systemically be applied, renew the progenitor cell pool, and fuse with existing myotubes to form regenerating fibers, thereby preventing or relieving muscle symptoms in patients with DM1.

MATERIALS AND METHODS

Ethics Statement

Animal experiments were approved by the Animal Ethics Committee of Radboud University (permit no. RU-DEC 2016-0049). The human biopsy procedure including the cell culture protocol was approved by the local Committee on Research Involving Human Subjects (CMO 2016-0049). Informed consent to participate in our study was obtained from all participants. No adverse events following the biopsy procedure were reported.

DMSXL Mice

DMSXL mice (C57BL/6 background) used in this study carry a 45-kb genomic fragment encompassing the DM1 locus of a patient, including a (CTG)1300 repeat.[28,29,56] Mouse number 4, 5, 11, and 12 were females; all others were males. Transgenic status was assessed by PCR on ear punch DNA. The DMSXL phenotype includes reduced muscle strength, lower motor performances, and respiratory impairment, which is essentially only apparent in HOM mice. On the molecular level, weak splicing abnormalities and RNA foci are present in skeletal muscle from HOM mice.[30,57]

Human Participants

DM1 patients and unaffected controls visited Radboudumc (Nijmegen, the Netherlands) where a skeletal muscle biopsy was taken from the *quadriceps femoris* (*vastus lateralis*) using a Bergström needle.[58] Specifications of the eight participants can be found in Table 1. MIRS was the latest score of clinical evaluation.[31] (CTG)n repeat length was determined in blood DNA at diagnosis of the disease.

Cell Culture Media

Aliquots of 80 mL fetal calf serum (FCS, Sigma-Aldrich) were heat inactivated in a water bath at 56°C for 40 min. Horse serum (HS, Sigma-Aldrich) was aliquoted into 10 mL and heat-inactivated at 56°C for 30 min. Proliferation medium for mouse pericytes (DMEM20) was made by combining DMEM (AQmedia, Sigma-Aldrich) with 20% heat-inactivated FCS, 50 IU/mL penicillin, 0.05 mg/mL streptomycin (Sigma-Aldrich), 10 mM nonessential amino acids (Sigma-Aldrich), 1 mM sodium pyruvate, and 50 mM β-mercaptoethanol (Gibco) over a 0.22-μm filter. Proliferation medium for human pericytes (DMEM5) has a similar composition but without sodium pyruvate, only 5% heat-inactivated FCS, and an additional 5 ng/mL human basic fibroblast growth factor (Sigma-Aldrich). Differentiation medium contained DMEM with 2% heat-inactivated horse serum, 50 IU/mL penicillin, 0.05 mg/mL streptomycin, and 1 mM sodium pyruvate. Coating of cell culture surfaces was performed with collagen from calf skin (Sigma-Aldrich) at 1 mg/mL dissolved in 20% glacial acetic acid. Then, 1 mg/mL collagen (20% glacial acid) was applied to the surface and left for at least 15 min at room temperature. The solution was removed and the collagen-coated wells and/or flasks were transferred to a dedicated 30°C oven to dry the surface for 24 h. The next day, the surface was washed twice with 1× PBS and once with a medium containing phenol red to ensure no glacial acid was left behind.

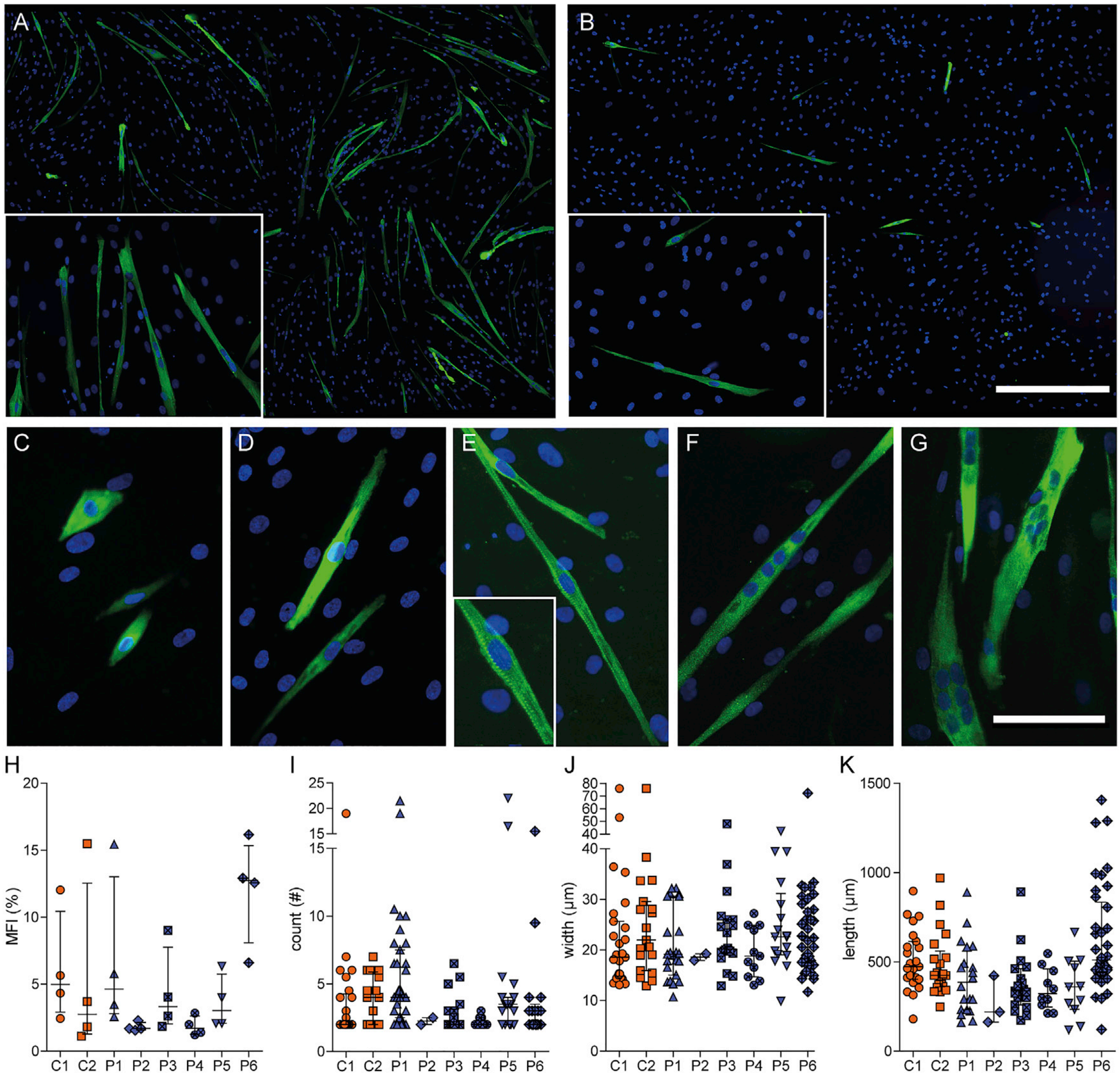

Figure 7. Myogenic Potential of DM1 Patient and Control Pericytes

Human pericytes were cultured under low-serum conditions and stained for differentiation marker MHC (green) and DNA (blue). Overview of a relatively efficient (A) and inefficient (B) differentiation culture. Scale bar in (A) and (B), 500 μm. (C–G) Typical cell morphologies and features that were observed in the cultures: roundish (C) and elongated (D) mononuclear cells; mononuclear cells with typical striated sarcomeric features (E); long multinucleated myotube (F); multinucleated myosac (G). Scale bar in (C)–(G), 100 μm. Differentiation cultures of each pericyte isolation were separately analyzed for MFI (H), number of nuclei per myotube (I), myotube width (J), and myotube length (K). Each data point represents the average MFI per culture (n = 4) (H), the number of nuclei per one myotube (n = 2–35) (I), width of one myotube (n = 2–38) (J), and the length (n = 3–37) (K). Bars indicate median with interquartile range.

Human and Mouse Explant Cultures

DMSXL mice were euthanized via CO_2 and weighed. Skeletal muscles of both hind limbs (GPS complex [gastrocnemius-plantaris-soleus complex]) of 4- to 5-week-old mice were dissected, weighed, and collected in DMEM20 with as little manipulations as possible to increase the yield of cells. Tissue from human participants was collected in 1× PBS on ice. For both mouse and human skeletal muscle biopsies, the following procedures were performed in a laminar flow hood. Muscle tissue was transferred into a 10-cm dish containing the appropriate medium. Any residues of fat or tendon were carefully removed while the tissue was dissected into ~2-mm^3 fragments with a sterile round-shaped scalpel. For human material, around half of the muscle biopsy already consisted of small fragments due to the collection procedure. To avoid contamination with blood as much as possible, tissue fragments were washed repeatedly with 1× PBS. Around 12 muscle fragments were then placed per well in a collagen coated six-well plate. To ensure maximal yield, the muscle fragments were distributed over the surface of the well. 200 μL of prewarmed DMEM5 (human explant) or DMEM20 (mouse explant) was placed on top of all fragments in one well, and an additional 400 μL was

dropwise added to the six-well plate. MQ (milliQ [ultrapure water]) was added to the space between wells to ensure a humidified environment and keep the tissue from drying out and detaching. Explants were incubated at 37°C for 18–24 h in a 5% CO_2/5% O_2 incubator. The next day, 1.5 mL prewarmed DMEM20 or DMEM5 medium was slowly added to the side of the well to avoid detachment. Explant cultures were then left undisturbed for 2–3 days. Remaining erythrocytes started to sprout, after which elongated, attached fibroblast-like cells migrated from the fragment, followed by pericytes. Cells initially looked round and small and then attached to the surface. After 4–5 days for mouse explants (7–8 days for human explants), the outgrowth of pericytes was visible and an additional 500 μL of prewarmed medium was added. From here onward fragments were monitored daily to inspect the extent of cell outgrowth from the explants. After 6–7 days for mouse explants (14–15 days for human explants), a 1-cm outgrowth of cells in a wavy pattern in all wells was present, surrounding almost every muscle fragment in the well. Cells were collected by trypsinization for FACS.

FACS

Media and harvested cells were collected over a 70-μm cell strainer to separate cells from the explants. Cells were counted and subsequently resuspended in 200 μL staining medium, i.e., HBSS Ca^{2+} Mg^{2+} (Sigma-Aldrich) supplemented with 2% heat-inactivated FCS and 10 mM HEPES (Sigma-Aldrich). Staining was performed with phycoerythrin-conjugated monoclonal anti-human/mouse ALP, clone B4-78 (R&D Systems), at 10 μl/10^6 cells and 0.3 μl per sample of viability dye eFluor 780 (eBioscience) for 45 min at 4°C in the dark. Control samples and FACS samples (ideally >1 × 10^6) were prepared in sterile FACS-suitable capped polystyrene tubes. Thereafter, the cells were washed three times with 1× PBS and filtered over a 70-μm filter to be ready for cell sorting. Cell sorting was performed on a BD FACSAria flow cytometer (BD Biosciences). Gates were set on fluorescence minus one (FMO) (human samples) and negative controls (mouse samples). ALP^+ pericytes were collected in the appropriate media. Collected cells were cultured at 37°C in a 5% CO_2/5% O_2 humidified incubator. Cell cultures were passaged upon 70%–80% confluence at a 1:4 ratio. Cells were disassociated by Tryple Express (Invitrogen) into a single-cell suspension and replated on collagen-coated wells/flasks.

Characterization of Pericytes

Cells were seeded on glass coverslips coated with calf skin collagen. Coated coverslips were washed with 1× PBS, and 10,000–15,000 cells per cm^2 were seeded. One day later, coverslips were washed with 1× PBS at room temperature. Fixation methods varied between conditions. Myotube cultures were fixed with 2% paraformaldehyde (PFA) in 0.1 M phosphate buffer (pH 7.4) for 15 min at RT. For immunodetection of NG2 (ab129051, Abcam), α-SMA (ab7817, Abcam) and MHC (MF20 DSHB, University of Iowa) cells were fixed in 4% PFA at RT for 10 min. In case of immunodetection of PDGFRβ (MA5-15143, Thermo Fisher), cells were fixed in 1:1 acetone/methanol for 10 min at −20°C. Cells were subsequently washed three times for 5 min with 1× PBS, after which they were permeabilized with 0.2% Triton X-100 (Sigma-Aldrich) for 10 min. Cells were incubated with blocking buffer (0.1% Triton X-100 [Sigma-Aldrich], 4% normal goat serum [NGS, Sigma-Aldrich], and 0.1% BSA [Sigma-Aldrich] in 1× PBS) for 60 min. Incubation with primary antibodies was done in blocking buffer, overnight at 4°C. Cells were then washed again three times with 1× PBS and incubated with 4 μg/mL goat anti-mouse Alexa Fluor 568 or goat anti-rabbit Alexa Fluor 488 (Thermo Fisher) and 100 ng/mL 4′,6-diamidino-2-phenylindole (DAPI) (Sigma-Aldrich) in blocking buffer for 60 min at RT in the dark. Cells were washed with 1× PBS and mounted on slides. Fluorescent images were acquired with a Leica DMI6000B microscope using a ×63 objective.

For enzymatic ALP staining, cells were washed with 1× PBS, fixed for 5 min with 4% PFA, and washed again with 1× PBS before being covered with BCIP/NBT solution (B1911, Sigma-Aldrich). Images were acquired with the ZOE fluorescent cell imager (Bio-Rad Laboratories).

Analysis of Myotube Characteristics and Image Analysis

To assess myogenic capacity via MHC staining, 30,000 pericytes were seeded in eight-well ibidi chambers coated with 1 mg/mL collagen in 20% glacial acetic acid and propagated for 2 days in growth medium until 100% confluency. Culturing in differentiation medium continued for 8 more days. Cells were washed with 1× PBS and fixed with 2% PFA in 0.1 M phosphate buffer for 15 min at RT. After fixation, cells were washed three times with 1× PBS and permeabilized for 10 min with 0.2% Triton X-100 in 1× PBS. Blocking was performed at RT for 1 h with blocking buffer containing 4% NGS (Sigma-Aldrich), 0.1% Triton X-100 (Sigma-Aldrich), and 0.1% BSA (Sigma-Aldrich). Samples were incubated overnight at 4°C with antibody dilution buffer with triton (ADB-T) containing 1:20 diluted anti-MHC antibody (MF20 DSHB, University of Iowa). Cells were washed three times with 1× PBS and incubated with goat anti-mouse Alexa Fluor 488 and 100 ng/mL DAPI (Sigma-Aldrich) in blocking buffer for 1 h at RT in the dark. Cells were washed three times with 1× PBS and stored in 1× PBS. Fluorescent images were acquired using a Leica DMI6000B microscope with a ×20 objective. Four-by-four tile scans were made to fit the large myotubes within the images.

Fiji software (ImageJ v1.52d) was used for analysis of myotube formation. The myogenic fusion index (MFI) was calculated as 100 × the ratio of the number of nuclei inside MHC^+ myotubes to the number of total nuclei. The number of DAPI-positive nuclei in myotubes was determined by counting the number of nuclei that co-localized with positive MHC staining using the multipoint tool of ImageJ. Total nuclei number was determined by autothresholding the DAPI channel with Huang, applying a watershed on the binary image to separate overlaying nuclei and automatic cell count using analyze particles (settings: 200 to infinity for pixel size). Using the Bezier curve tool, myotube length and width were determined. The longest myotube branch was measured while the broadest part of the myotubes was measured to determine the average width. For these measurements, 20 myotubes per sample were measured from two independent experiments. All quantifications were performed in duplicate by two independent researchers.

qRT-PCR

In human samples, *DMPK* expression levels were determined at passage 8. To quantify expression levels in cells from WT, HET, and HOM DMSXL mice, qRT-PCR was performed in three biological replicates (three mice per genotype) and two technical replicates. Cell pellets of passage 5–7 were collected and stored in 350 μL of RA1 lysis buffer (Macherey-Nagel NucleoSpin RNA, lot no. 75858). RNA isolation for all samples was performed using the NucleoSpin RNA isolation kit (Macherey-Nagel) according to the manufacturer's protocol. Next, cDNA synthesis with random hexamers from the iScript cDNA synthesis kit (Bio-Rad Laboratories) was performed using 2 μg of RNA as a template in a reaction volume of 40 μL. For qRT-PCR, cDNA was diluted 40×. The cDNA sample was mixed in a final volume of 25 μL containing GoTaq qPCR Master Mix, distilled H_2O, and 0.4 pmol of each primer. Samples were analyzed using the 7900HT Fast Real-Time PCR System (Thermo Fisher). RT minus and no-template control were included as negative controls. A DM500 myoblast RNA sample[17] was included as a positive control for analysis of *Dmpk* expression, while DM11 cells[50] were included as a reference for *DMPK* expression. Gene-of-interest levels were normalized to *Gapdh* and *Hprt1* levels for mouse pericytes. *TBP* and *HPRT1* were included as reference genes for human pericytes.

PCR primers used were as follows: *Gapdh* e2–e3, 5′-CGTAGACAAAATGGTGAAG-3′ and 5′-GTTGATGGCAACAATCTC-3′; *Hprt1* e6–e7, 5′-GACACTGGTAAAACAATGC-3′ and 5′-CTGTATCCAACACTTCGAG-3′; *Dmpk* e2–e3, 5′-TTTTGAAGGTGATCGGGCGTG-3′ and 5′-CCTCTCTTCAGCATGTCCCACTTA-3′; *DMPK* e15-3′, 5′-TGCCTGCTTACTCGGGAAA-3′ and 5′-GAGCAGCGCAAGTGAGGAG-3′; *TBP* e3, 5′-CCACTCACAGACTCTCACAAC-3′ and 5′-CTGCGGTACAATCCCAGAACT-3′; *HPRT1* e7–e9, 5′-TGACAGTGGCAAAACAATG-3′ and 5′-GGTCCTTTTCACCAGCAAGCT-3′.

RNA FISH and Image Analysis

Mouse and human pericytes were grown on coverslips, which were coated with 1 mg/mL collagen in 20% glacial acetic acid, until 40%–50% confluency. The cells were washed with 1× PBS and fixed in 4% PFA for 10 min at RT. After washing three times for 5 min with 1× PBS, coverslips were prehybridized in 40% deionized formamide (Ambion) in 2× standard saline citrate (saline sodium citrate [SSC]; Ambion) for 20 min at RT. Overnight hybridization was performed at 37°C with 0.1 ng/mL TYE563-labeled 5′-CAGCAGCAGCAGCAGCAG-3′ locked nucleic acid (LNA) probe (Exiqon) in hybridization buffer containing 40% deionized formamide, 2 mg/mL BSA, 100 mg/mL dextran sulfate (Pharmacia), 0.1% Triton X-100, 1 mg/mL herring sperm DNA (Promega), 100 mg/mL yeast transfer RNA (Ambion), and 2 mM vanadyl ribonucleoside complex (New England BioLabs) in 2× SSC. Coverslips were washed twice for 5 min with 1× PBS before counterstaining cell nuclei with 100 ng/mL DAPI in 1× PBS for 10 min at RT. Cells were then washed twice for 5 min with 1× PBS, and coverslips were mounted with Mowiol 4-88 (Sigma-Aldrich). Fluorescent images for quantification of foci in mouse pericytes were acquired using a Leica DMI6000B microscope with a ×63 objective. Images were subsequently analyzed using Fiji software. DAPI masks were created using Yen's auto threshold followed by a watershed. The "find maxima" option in Fiji was applied for the tetramethylrhodamine isothiocyanate channel using a noise tolerance of 250, resulting in images containing single points. Using the DAPI masks, the positive pixels representing the foci in each nucleus were counted.

Fluorescent images for quantification of human pericytes were acquired using a Zeiss LSM880 with Airyscan. Moreover, to visualize co-accumulation of MBNL1 protein with (CUG)n-expanded *DMPK* transcripts in nuclear foci, coverslips were fixed in acetone/methanol (1:1) for 5 min at −20°C. After washing three times for 5 min with 1× PBS, 0.1% Triton X-100 in 1× PBS was applied for 10 min, after which blocking buffer (3% BSA, 0.1% glycine [Merck], and 0.1% Triton X-100) was applied for 1 h at RT. Samples were then incubated overnight at 4°C in the same buffer containing 1:10 diluted anti-MBNL1 antibody, MB1a (4A8; DSHB, University of Iowa). The next day, samples were washed three times for 5 min with 1× PBS and incubated with 4 mg/mL goat anti-mouse Alexa Fluor 488 (Thermo Fisher) and 100 ng/mL DAPI in blocking buffer for 1 h at room temperature in the dark. Finally, coverslips were washed with PBS, Milli-Q, and 70% and 100% ethanol and mounted with Mowiol 4-88 (Sigma-Aldrich). Images from the Zeiss LSM880 with Airyscan were analyzed using Fiji software. Nuclear masks were created using Isodata auto-threshold followed by a watershed and particle analysis ("size=1000-Infinity pixel" for the FISH only and "size=500-Infinity pixel" for the FISH-MBNL1 analysis). Within the nuclear selection, the "spot counter" in Fiji was applied using a noise tolerance of 50 for FISH only and a noise tolerance of 120 for the FISH-MBNL1 analysis. Using the nuclear masks, the positive pixels representing the foci in each nucleus were counted. For MBNL1 co-localization, a region of interest (ROI) was established around each FISH focus. The signal outside was cleared, and only the ROI with a FISH focus and MBNL1 signal were quantified using the macros "find maxima single point" and "spot counter."

SUPPLEMENTAL INFORMATION

Supplemental Information can be found online at https://doi.org/10.1016/j.omtm.2019.09.002.

AUTHOR CONTRIBUTIONS

Conceptualization, C.R.M.A., R.H.L.R., D.G.W., B.G.M.v.E., and H.v.B.; Investigation, C.R.M.A., R.H.L.R., and M.W.; Formal Analysis, C.R.M.A., R.H.L.R., W.J.A.A.v.d.B., and M.W.; Writing – Original Draft, C.R.M.A, R.H.L.R, M.W., and D.G.W.; Writing – Review and Editing, C.R.M.A, R.H.L.R, W.J.A.A.v.d.B., D.G.W., B.G.M.v.E, and H.v.B.; Visualization, C.R.M.A, R.H.L.R, M.W., and D.G.W.; Supervision, D.G.W., B.G.M.v.E, and H.v.B.

CONFLICTS OF INTEREST

The authors declare no competing interests.

ACKNOWLEDGMENTS

We are indebted to all participants who engaged in this research and made it possible, and to Joost Raaphorst, Fran Smulders, and Yvonne Cornelissen (Nijmegen, Netherlands) who assisted with recruitment and collection of human muscle biopsy material. We are grateful to Prof. Maurilio Sampaolesi (Leuven, Belgium) and Florence van Tienen (Maastricht, Netherlands), who taught us the technique of pericyte isolation. We thank Prof. Geneviève Gourdon (Paris, France) for the DM500 and DMSXL mouse lines. We thank the members of the department of Cell Biology, especially Prof. Bé Wieringa, for helpful discussions and advice. This work was funded by the Donders lnstitute for Brain Cognition and Behavior, Radboud University Medical Center, and supported by the Prinses Beatrix Spierfonds (grant W.OR18-18; with contribution from the Stichting Spieren voor Spieren).

REFERENCES

1. De Angelis, L., Berghella, L., Coletta, M., Lattanzi, L., Zanchi, M., Cusella-De Angelis, M.G., Ponzetto, C., and Cossu, G. (1999). Skeletal myogenic progenitors originating from embryonic dorsal aorta coexpress endothelial and myogenic markers and contribute to postnatal muscle growth and regeneration. J. Cell Biol. *147*, 869–878.
2. Minasi, M.G., Riminucci, M., De Angelis, L., Borello, U., Berarducci, B., Innocenzi, A., Caprioli, A., Sirabella, D., Baiocchi, M., De Maria, R., et al. (2002). The meso-angioblast: a multipotent, self-renewing cell that originates from the dorsal aorta and differentiates into most mesodermal tissues. Development *129*, 2773–2783.
3. Dellavalle, A., Sampaolesi, M., Tonlorenzi, R., Tagliafico, E., Sacchetti, B., Perani, L., Innocenzi, A., Galvez, B.G., Messina, G., Morosetti, R., et al. (2007). Pericytes of human skeletal muscle are myogenic precursors distinct from satellite cells. Nat. Cell Biol. *9*, 255–267.
4. Birbrair, A., and Delbono, O. (2015). Pericytes are essential for skeletal muscle formation. Stem Cell Rev. Rep. *11*, 547–548.
5. Kostallari, E., Baba-Amer, Y., Alonso-Martin, S., Ngoh, P., Relaix, F., Lafuste, P., and Gherardi, R.K. (2015). Pericytes in the myovascular niche promote post-natal myofiber growth and satellite cell quiescence. Development *142*, 1242–1253.
6. Dellavalle, A., Maroli, G., Covarello, D., Azzoni, E., Innocenzi, A., Perani, L., Antonini, S., Sambasivan, R., Brunelli, S., Tajbakhsh, S., and Cossu, G. (2011). Pericytes resident in postnatal skeletal muscle differentiate into muscle fibres and generate satellite cells. Nat. Commun. *2*, 499.
7. Berry, S.E. (2015). Concise review: mesoangioblast and mesenchymal stem cell therapy for muscular dystrophy: progress, challenges, and future directions. Stem Cells Transl. Med. *4*, 91–98.
8. Sampaolesi, M., Blot, S., D'Antona, G., Granger, N., Tonlorenzi, R., Innocenzi, A., Mognol, P., Thibaud, J.L., Galvez, B.G., Barthélémy, I., et al. (2006). Mesoangioblast stem cells ameliorate muscle function in dystrophic dogs. Nature *444*, 574–579.
9. Galvez, B.G., Sampaolesi, M., Brunelli, S., Covarello, D., Gavina, M., Rossi, B., Constantin, G., Torrente, Y., and Cossu, G. (2006). Complete repair of dystrophic skeletal muscle by mesoangioblasts with enhanced migration ability. J. Cell Biol. *174*, 231–243.
10. Morosetti, R., Gidaro, T., Broccolini, A., Gliubizzi, C., Sancricca, C., Tonali, P.A., Ricci, E., and Mirabella, M. (2011). Mesoangioblasts from facioscapulohumeral muscular dystrophy display in vivo a variable myogenic ability predictable by their in vitro behavior. Cell Transplant. *20*, 1299–1313.
11. Quattrocelli, M., Palazzolo, G., Perini, I., Crippa, S., Cassano, M., and Sampaolesi, M. (2012). Mouse and human mesoangioblasts: isolation and characterization from adult skeletal muscles. Methods Mol. Biol. *798*, 65–76.
12. Mateos-Aierdi, A.J., Goicoechea, M., Aiastui, A., Fernández-Torrón, R., Garcia-Puga, M., Matheu, A., and López de Munain, A. (2015). Muscle wasting in myotonic dystrophies: a model of premature aging. Front. Aging Neurosci. *7*, 125.
13. Wertz, R.L., Hartwig, G.B., Frost, A.P., Brophy, J.J., Atwater, S.K., and Roses, A.D. (1981). Patients with myotonic dystrophy, a possible segmental progeroid syndrome, and Duchenne muscular dystrophy have fibroblasts with normal limits for in vitro lifespan and growth characteristics. J. Cell. Physiol. *107*, 255–260.
14. Rotini, A., Martínez-Sarrà, E., Duelen, R., Costamagna, D., Di Filippo, E.S., Giacomazzi, G., Grosemans, H., Fulle, S., and Sampaolesi, M. (2018). Aging affects the in vivo regenerative potential of human mesoangioblasts. Aging Cell. Published online February 4, 2018. https://doi.org/10.1111/acel.12714.
15. Mulders, S.A., van Engelen, B.G., Wieringa, B., and Wansink, D.G. (2010). Molecular therapy in myotonic dystrophy: focus on RNA gain-of-function. Hum. Mol. Genet. *19*, R90–R97.
16. Gudde, A.E.E.G., van Heeringen, S.J., de Oude, A.I., van Kessel, I.D.G., Estabrook, J., Wang, E.T., Wieringa, B., and Wansink, D.G. (2017). Antisense transcription of the myotonic dystrophy locus yields low-abundant RNAs with and without (CAG)n repeat. RNA Biol. *14*, 1374–1388.
17. Gudde, A.E., González-Barriga, A., van den Broek, W.J., Wieringa, B., and Wansink, D.G. (2016). A low absolute number of expanded transcripts is involved in myotonic dystrophy type 1 manifestation in muscle. Hum. Mol. Genet. *25*, 1648–1662.
18. Pearson, C.E., Nichol Edamura, K., and Cleary, J.D. (2005). Repeat instability: mechanisms of dynamic mutations. Nat. Rev. Genet. *6*, 729–742.
19. Mirkin, S.M. (2007). Expandable DNA repeats and human disease. Nature *447*, 932–940.
20. Zu, T., Gibbens, B., Doty, N.S., Gomes-Pereira, M., Huguet, A., Stone, M.D., Margolis, J., Peterson, M., Markowski, T.W., Ingram, M.A., et al. (2011). Non-ATG-initiated translation directed by microsatellite expansions. Proc. Natl. Acad. Sci. USA *108*, 260–265.
21. Zhang, K., Daigle, J.G., Cunningham, K.M., Coyne, A.N., Ruan, K., Grima, J.C., Bowen, K.E., Wadhwa, H., Yang, P., Rigo, F., et al. (2018). Stress granule assembly disrupts nucleocytoplasmic transport. Cell *173*, 958–971.e17.
22. Udd, B., and Krahe, R. (2012). The myotonic dystrophies: molecular, clinical, and therapeutic challenges. Lancet Neurol. *11*, 891–905.
23. Jansen, G., Groenen, P.J., Bächner, D., Jap, P.H., Coerwinkel, M., Oerlemans, F., van den Broek, W., Gohlsch, B., Pette, D., Plomp, J.J., et al. (1996). Abnormal myotonic dystrophy protein kinase levels produce only mild myopathy in mice. Nat. Genet. *13*, 316–324.
24. Harmon, E.B., Harmon, M.L., Larsen, T.D., Paulson, A.F., and Perryman, M.B. (2008). Myotonic dystrophy protein kinase is expressed in embryonic myocytes and is required for myotube formation. Dev. Dyn. *237*, 2353–2366.
25. Tedesco, F.S., Dellavalle, A., Diaz-Manera, J., Messina, G., and Cossu, G. (2010). Repairing skeletal muscle: regenerative potential of skeletal muscle stem cells. J. Clin. Invest. *120*, 11–19.
26. Pierantozzi, E., Vezzani, B., Badin, M., Curina, C., Severi, F.M., Petraglia, F., Randazzo, D., Rossi, D., and Sorrentino, V. (2016). Tissue-specific cultured human pericytes: perivascular cells from smooth muscle tissue have restricted mesodermal differentiation ability. Stem Cells Dev. *25*, 674–686.
27. Valadares, M.C., Gomes, J.P., Castello, G., Assoni, A., Pellati, M., Bueno, C., Corselli, M., Silva, H., Bartolini, P., Vainzof, M., et al. (2014). Human adipose tissue derived pericytes increase life span in Utrn (tm1Ked) Dmd (mdx) /J mice. Stem Cell Rev Rep *10*, 830–840.
28. Seznec, H., Lia-Baldini, A.S., Duros, C., Fouquet, C., Lacroix, C., Hofmann-Radvanyi, H., Junien, C., and Gourdon, G. (2000). Transgenic mice carrying large human genomic sequences with expanded CTG repeat mimic closely the DM CTG repeat intergenerational and somatic instability. Hum. Mol. Genet. *9*, 1185–1194.
29. Gomes-Pereira, M., Foiry, L., Nicole, A., Huguet, A., Junien, C., Munnich, A., and Gourdon, G. (2007). CTG trinucleotide repeat "big jumps": large expansions, small mice. PLoS Genet. *3*, e52.
30. Huguet, A., Medja, F., Nicole, A., Vignaud, A., Guiraud-Dogan, C., Ferry, A., Decostre, V., Hogrel, J.Y., Metzger, F., Hoeflich, A., et al. (2012). Molecular, physiological, and motor performance defects in DMSXL mice carrying >1,000 CTG repeats from the human DM1 locus. PLoS Genet. *8*, e1003043.
31. Mathieu, J., Boivin, H., Meunier, D., Gaudreault, M., and Bégin, P. (2001). Assessment of a disease-specific muscular impairment rating scale in myotonic dystrophy. Neurology *56*, 336–340.

32. Nirwane, A., Gautam, J., and Yao, Y. (2017). Isolation of Type I and Type II Pericytes from Mouse Skeletal Muscles. J Vis. Exp. *123*, e55904.

33. Sicot, G., Gourdon, G., and Gomes-Pereira, M. (2011). Myotonic dystrophy, when simple repeats reveal complex pathogenic entities: new findings and future challenges. Hum. Mol. Genet. *20*, R116–R123.

34. André, L.M., Ausems, C.R.M., Wansink, D.G., and Wieringa, B. (2018). Abnormalities in skeletal muscle myogenesis, growth, and regeneration in myotonic dystrophy. Front. Neurol. *9*, 368.

35. Gladman, J.T., Mandal, M., Srinivasan, V., and Mahadevan, M.S. (2013). Age of onset of RNA toxicity influences phenotypic severity: evidence from an inducible mouse model of myotonic dystrophy (DM1). PLoS ONE *8*, e72907.

36. Tonlorenzi, R., Dellavalle, A., Schnapp, E., Cossu, G., and Sampaolesi, M. (2007). Isolation and characterization of mesoangioblasts from mouse, dog, and human tissues. Curr. Protoc. Stem Cell Biol. *3*, 2B.1.1–2B.1.29.

37. Safadi, A., Livne, E., Silbermann, M., and Reznick, A.Z. (1991). Activity of alkaline phosphatase in rat skeletal muscle localized along the sarcolemma and endothelial cell membranes. J. Histochem. Cytochem. *39*, 199–203.

38. Murakami, S., Fujino, H., Takeda, I., Momota, R., Kumagishi, K., and Ohtsuka, A. (2010). Comparison of capillary architecture between slow and fast muscles in rats using a confocal laser scanning microscope. Acta Med. Okayama *64*, 11–18.

39. Thornell, L.E., Lindstöm, M., Renault, V., Klein, A., Mouly, V., Ansved, T., Butler-Browne, G., and Furling, D. (2009). Satellite cell dysfunction contributes to the progressive muscle atrophy in myotonic dystrophy type 1. Neuropathol. Appl. Neurobiol. *35*, 603–613.

40. van Dijk, C.G., Nieuweboer, F.E., Pei, J.Y., Xu, Y.J., Burgisser, P., van Mulligen, E., el Azzouzi, H., Duncker, D.J., Verhaar, M.C., and Cheng, C. (2015). The complex mural cell: pericyte function in health and disease. Int. J. Cardiol. *190*, 75–89.

41. Hughes, S., Gardiner, T., Hu, P., Baxter, L., Rosinova, E., and Chan-Ling, T. (2006). Altered pericyte-endothelial relations in the rat retina during aging: implications for vessel stability. Neurobiol. Aging *27*, 1838–1847.

42. Murray, I.R., Baily, J.E., Chen, W.C.W., Dar, A., Gonzalez, Z.N., Jensen, A.R., Petrigliano, F.A., Deb, A., and Henderson, N.C. (2017). Skeletal and cardiac muscle pericytes: functions and therapeutic potential. Pharmacol. Ther. *171*, 65–74.

43. Hellström, M., Kalén, M., Lindahl, P., Abramsson, A., and Betsholtz, C. (1999). Role of PDGF-B and PDGFR-beta in recruitment of vascular smooth muscle cells and pericytes during embryonic blood vessel formation in the mouse. Development *126*, 3047–3055.

44. Hughes, S., and Chan-Ling, T. (2004). Characterization of smooth muscle cell and pericyte differentiation in the rat retina in vivo. Invest. Ophthalmol. Vis. Sci. *45*, 2795–2806.

45. Morales, F., Vásquez, M., Cuenca, P., Campos, D., Santamaría, C., Del Valle, G., Brian, R., Sittenfeld, M., and Monckton, D.G. (2015). Parental age effects, but no evidence for an intrauterine effect in the transmission of myotonic dystrophy type 1. Eur. J. Hum. Genet. *23*, 646–653.

46. Larsson, C., Grundberg, I., Söderberg, O., and Nilsson, M. (2010). In situ detection and genotyping of individual mRNA molecules. Nat. Methods *7*, 395–397.

47. Ishikawa, K., Yoshida, K., Kanie, K., Omori, K., and Kato, R. (2019). Morphology-based analysis of myoblasts for prediction of myotube formation. SLAS Discov. *24*, 47–56.

48. Morosetti, R., Gliubizzi, C., Broccolini, A., Sancricca, C., and Mirabella, M. (2011). Mesoangioblasts of inclusion-body myositis: a twofold tool to study pathogenic mechanisms and enhance defective muscle regeneration. Acta Myol. *30*, 24–28.

49. Morosetti, R., Mirabella, M., Gliubizzi, C., Broccolini, A., Sancricca, C., Pescatori, M., Gidaro, T., Tasca, G., Frusciante, R., Tonali, P.A., et al. (2007). Isolation and characterization of mesoangioblasts from facioscapulohumeral muscular dystrophy muscle biopsies. Stem Cells *25*, 3173–3182.

50. van Agtmaal, E.L., André, L.M., Willemse, M., Cumming, S.A., van Kessel, I.D.G., van den Broek, W.J.A.A., Gourdon, G., Furling, D., Mouly, V., Monckton, D.G., et al. (2017). CRISPR/Cas9-induced $(CTG\cdot CAG)_n$ repeat instability in the myotonic dystrophy type 1 locus: implications for therapeutic genome editing. Mol. Ther. *25*, 24–43.

51. Larsen, J., Pettersson, O.J., Jakobsen, M., Thomsen, R., Pedersen, C.B., Hertz, J.M., Gregersen, N., Corydon, T.J., and Jensen, T.G. (2011). Myoblasts generated by lentiviral mediated MyoD transduction of myotonic dystrophy type 1 (DM1) fibroblasts can be used for assays of therapeutic molecules. BMC Res. Notes *4*, 490.

52. Dastidar, S., Ardui, S., Singh, K., Majumdar, D., Nair, N., Fu, Y., Reyon, D., Samara, E., Gerli, M.F.M., Klein, A.F., et al. (2018). Efficient CRISPR/Cas9-mediated editing of trinucleotide repeat expansion in myotonic dystrophy patient-derived iPS and myogenic cells. Nucleic Acids Res. *46*, 8275–8298.

53. Péault, B., Rudnicki, M., Torrente, Y., Cossu, G., Tremblay, J.P., Partridge, T., Gussoni, E., Kunkel, L.M., and Huard, J. (2007). Stem and progenitor cells in skeletal muscle development, maintenance, and therapy. Mol. Ther. *15*, 867–877.

54. Sampaolesi, M., Torrente, Y., Innocenzi, A., Tonlorenzi, R., D'Antona, G., Pellegrino, M.A., Barresi, R., Bresolin, N., De Angelis, M.G., Campbell, K.P., et al. (2003). Cell therapy of α-sarcoglycan null dystrophic mice through intra-arterial delivery of mesoangioblasts. Science *301*, 487–492.

55. Cossu, G., Previtali, S.C., Napolitano, S., Cicalese, M.P., Tedesco, F.S., Nicastro, F., Noviello, M., Roostalu, U., Natali Sora, M.G., Scarlato, M., et al. (2015). Intra-arterial transplantation of HLA-matched donor mesoangioblasts in Duchenne muscular dystrophy. EMBO Mol. Med. *7*, 1513–1528.

56. Gourdon, G., Radvanyi, F., Lia, A.S., Duros, C., Blanche, M., Abitbol, M., Junien, C., and Hofmann-Radvanyi, H. (1997). Moderate intergenerational and somatic instability of a 55-CTG repeat in transgenic mice. Nat. Genet. *15*, 190–192.

57. Braz, S.O., Acquaire, J., Gourdon, G., and Gomes-Pereira, M. (2018). *Of Mice and Men:* advances in the understanding of neuromuscular aspects of myotonic dystrophy. Front. Neurol. *9*, 519.

58. Voermans, N.C., van Alfen, N., Pillen, S., Lammens, M., Schalkwijk, J., Zwarts, M.J., van Rooij, I.A., Hamel, B.C., and van Engelen, B.G. (2009). Neuromuscular involvement in various types of Ehlers-Danlos syndrome. Ann. Neurol. *65*, 687–697.

OMTM, Volume *15*

Supplemental Information

Intrinsic Myogenic Potential of Skeletal Muscle-Derived Pericytes from Patients with Myotonic Dystrophy Type 1

Cornelia Rosanne Maria Ausems, Renée Henrica Lamberta Raaijmakers, Walterus Johannes Antonius Adriana van den Broek, Marieke Willemse, Baziel Gerardus Maria van Engelen, Derick Gert Wansink, and Hans van Bokhoven

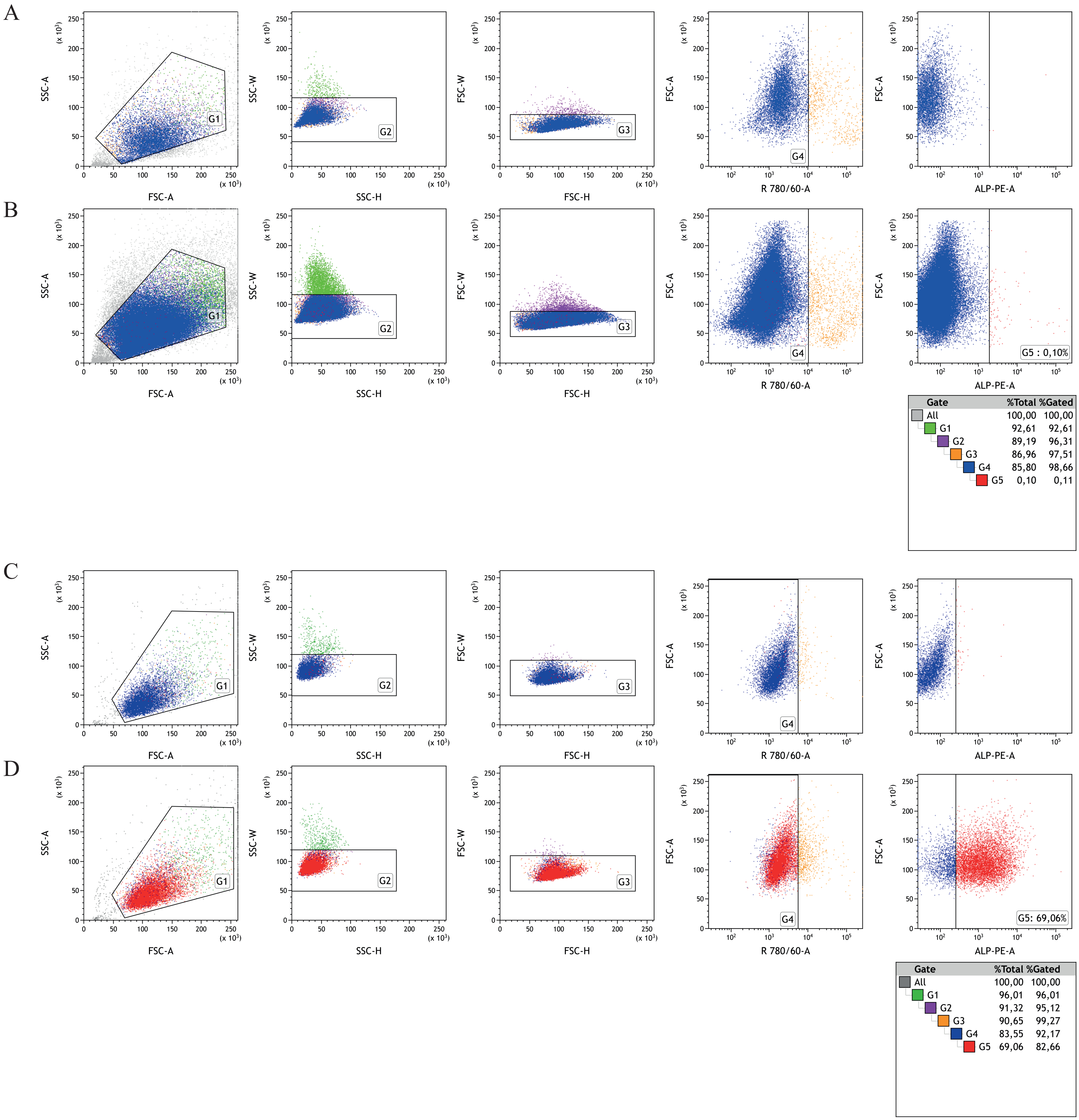

Figure S1. Gating boundaries and representative sorting of ALP⁺ pericytes from mouse and human explant cultures. Harvested cells from the explant cultures were sorted on ALP⁺. Cells in the fifth gate "G5" were considered ALP⁺ pericytes. The population hierarchy is shown under the plots. (A) Gating for all mouse samples was optimized according to a negative control without ALP⁻ phycoerythrin (PE). (B) Fluorescent plot of the sorting of mouse sample (M3), showing the distribution of the ALP-PE⁺ pericytes. (C) Gating for all human samples was optimized according to a negative control. (D) Fluorescent plot of the sorting of ALP-PE⁺ pericytes from the explant culture of P3. This supplemental figure relates to Figures 1 and 2.

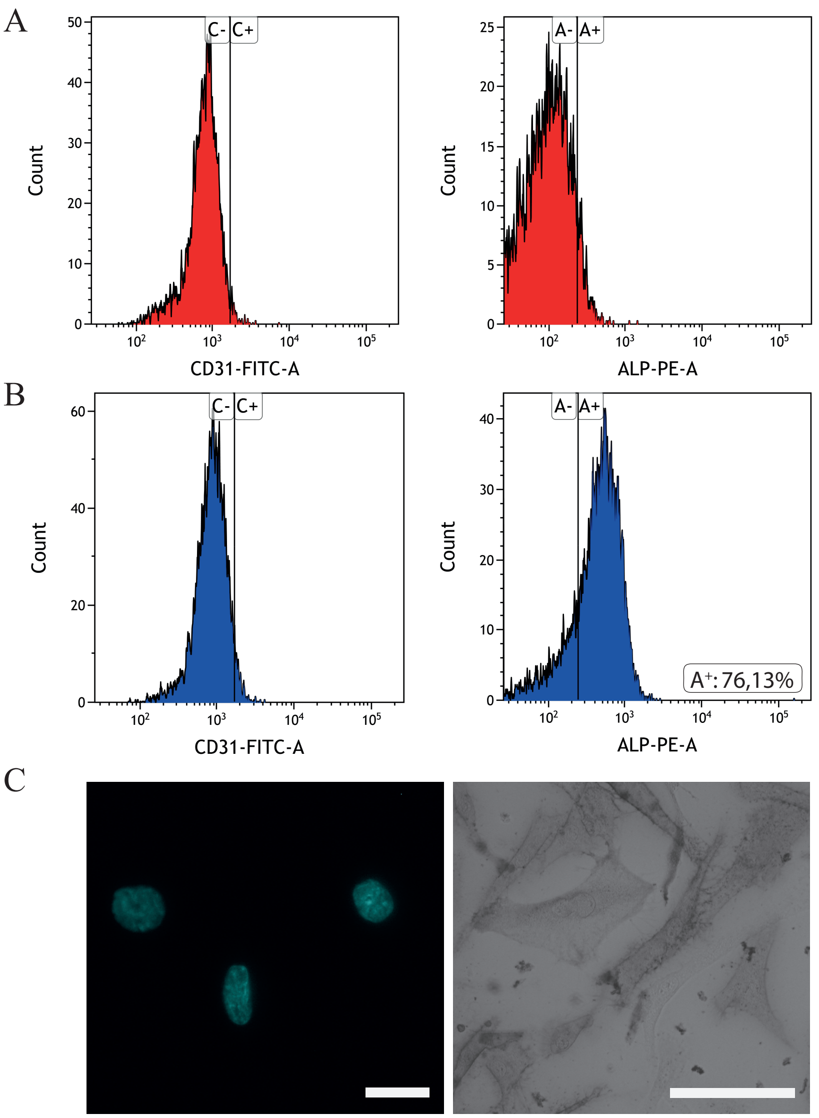

Figure S2. Determination of ALP expression in mouse and human pericytes after high cell passage number. Cell numbers of mouse pericytes plotted against CD31$^-$ fluorescein isothiocyanate (FITC) and ALP-PE staining. The input was a gated population without debris, cell doublets and dead cells (G4) (see Fig. S1B). A negative control without ALP-PE (A) was included to set the gate on background signal. The cell population remained ALP$^+$ after 19 passages (B). (C) Over time the culture of P6 showed stretched elongated cells. To verify whether at passage 20 the culture had started to differentiate, we stained for MHC expression (left) and ALP activity (right). All cells turned out to be MHC negative and ALP positive. Scale bars are 20 µm (left) and 100 µm (right). This supplemental figure relates to Figures 1 and 2.

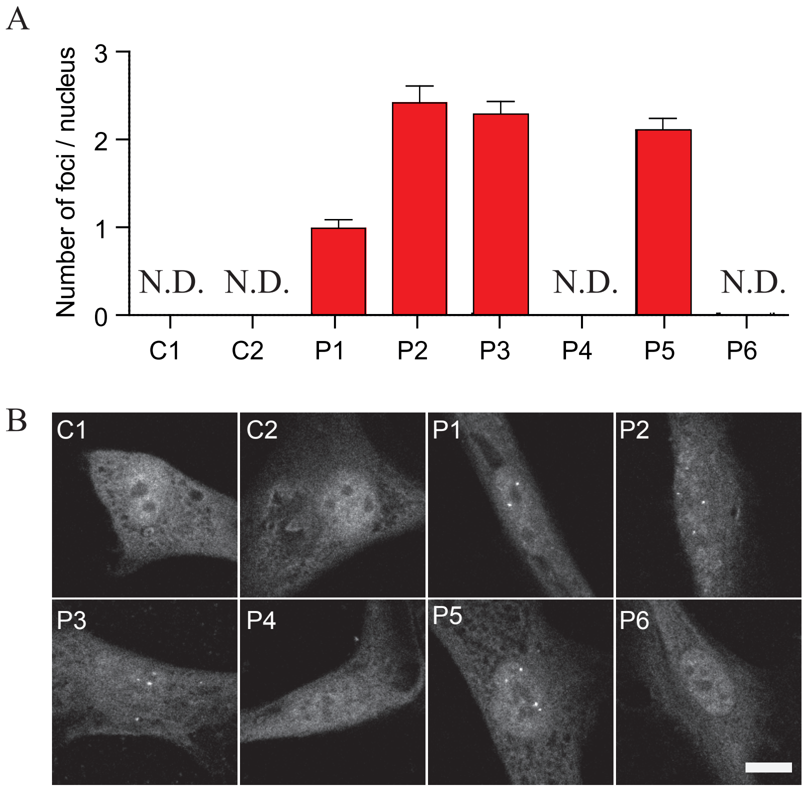

Figure S3. Representative RNA FISH images using a (CAG)7 oligonucleotide probe. Quantification of nuclear (CUG)n foci in pericytes isolated from two unaffected controls (C1, C2) and six DM1 patients (P1-P6). N.D. Not detectable. Scale bar is 10 µm. Error bars indicate SEM. This supplemental figure relates to Figure 6.

Table S1: Passage number and fusion index of pericyte cultures in myogenic differentiation experiments.

Participant number	Passage number experiment 1	Passage number experiment 2	MFI (%) experiment 1	MFI (%) experiment 2
C1	P04	P09	8.8	3.4
C2	P03	P05	2.8	10.5
P1	P01	P05	4.6	4.7
P2	P03	P04	1.9	1.7
P3	P02	P04	5.4	4.2
P4	P04	P04	1.6	1.6
P5	P04	P07	3.0	2.8
P6	P09	P12	14.5	9.6

MFI: myogenic fusion index
